# Heart failure with mildly reduced and preserved ejection fraction: A review of disease burden and remaining unmet medical needs within a new treatment landscape

**DOI:** 10.1007/s10741-024-10385-y

**Published:** 2024-02-27

**Authors:** Nihar Desai, Elzbieta Olewinska, Agata Famulska, Cécile Remuzat, Clément Francois, Kerstin Folkerts

**Affiliations:** 1 Section of Cardiovascular Medicine, Department of Internal Medicine, Yale School of Medicine, New Haven, CT USA; 2 Putnam, Cracow, Poland; 3 Putnam, Paris, France; 4 Bayer AG, Wuppertal, Germany

**Keywords:** Heart failure, HFpEF, Disease burden, Unmet needs, Epidemiology, Guidelines

## Abstract

This review provides a comprehensive overview of heart failure with mildly reduced and preserved ejection fraction (HFmrEF/HFpEF), including its definition, diagnosis, and epidemiology; clinical, humanistic, and economic burdens; current pharmacologic landscape in key pharmaceutical markets; and unmet needs to identify key knowledge gaps. We conducted a targeted literature review in electronic databases and prioritized articles with valuable insights into HFmrEF/HFpEF. Overall, 27 randomized controlled trials (RCTs), 66 real-world evidence studies, 18 clinical practice guidelines, and 25 additional publications were included. Although recent heart failure (HF) guidelines set left ventricular ejection fraction thresholds to differentiate categories, characterization and diagnosis criteria vary because of the incomplete disease understanding. Recent epidemiological data are limited and diverse. Approximately 50% of symptomatic HF patients have HFpEF, more common than HFmrEF. Prevalence varies with country because of differing definitions and study characteristics, making prevalence interpretation challenging. HFmrEF/HFpEF has considerable mortality risk, and the mortality rate varies with study and patient characteristics and treatments. HFmrEF/HFpEF is associated with considerable morbidity, poor patient outcomes, and common comorbidities. Patients require frequent hospitalizations; therefore, early intervention is crucial to prevent disease burden. Recent RCTs show promising results like risk reduction of composite cardiovascular death or HF hospitalization. Costs data are scarce, but the economic burden is increasing. Despite new drugs, unmet medical needs requiring new treatments remain. Thus, HFmrEF/HFpEF is a growing global healthcare concern. With improving yet incomplete understanding of this disease and its promising treatments, further research is required for better patient outcomes.

**Supplementary Information:**

The online version contains supplementary material available at 10.1007/s10741-024-10385-y.

## Background and objectives

Chronic heart failure (HF) is a multisystemic disorder and a leading cause of cardiovascular (CV) morbidity and mortality globally [1–3]. Its severity and impact on patients’ physical activity are typically categorized using the widely employed New York Heart Association (NYHA) functional classification system, which categorizes the severity of HF symptoms into classes I to IV. Class I refers to asymptomatic patients; class II, class III, and class IV correspond to mildly, moderately, and severely symptomatic patients, respectively [1, 2]. Furthermore, HF is classified into 3 groups based on patients’ left ventricular ejection fraction (LVEF): heart failure with reduced ejection fraction (HFrEF) (LVEF ≤ 40%); mildly reduced ejection fraction (HFmrEF) (LVEF 41%-49%); and preserved ejection fraction (HFpEF) (LVEF ≥ 50%) [1–3]. The prevalence of HF is increasing worldwide, largely because of aging populations and lifestyle factors contributing to a higher prevalence of risk factors [4, 5]. Data suggest that HFmrEF and HFpEF are projected to become the dominant HF subtypes in the future, given their substantial and growing prevalence among patients with HF worldwide, leading to substantial patient burden and unmet needs because of limited treatment options [4, 5]. Currently, only a few drugs have been specifically approved to treat HFmrEF/HFpEF. In recent years, initial approvals have been granted for sodium-glucose cotransporter-2 inhibitors (SGLT-2is)—including empagliflozin [6, 7] and dapagliflozin [8, 9]—and angiotensin receptor/neprilysin inhibitor (ARNI) sacubitril/valsartan [10]. Prior to these recent approvals, there was a lack of conclusive studies showing improvements in the course or prognosis of HFmrEF/HFpEF in the long term [1, 2, 5]. Previous reviews have presented data on the burden of illness and epidemiology of HFpEF and HFmrEF. However, some of these publications have relatively dated time frames or focus on countries out of key pharmaceutical markets like United States, Western Europe and Japan [11–13]. Considering the recent changes in the treatment landscape, the objective of this review is to provide the most up-to-date data on the definition and diagnosis, epidemiology, burden of illness, and current pharmacologic landscape in the United States, Europe (with a focus on the United Kingdom, France, Germany, and Sweden), and Japan in relation to HFmrEF/HFpEF. Finally, this review aims to assess the remaining unmet needs and identify key knowledge gaps.

## Methods

### Study design and search process

To assess the existing research on HFpEF and HFmrEF, a targeted literature review (TLR) was conducted. The search process adhered to the methods recommended by the Cochrane Collaboration Handbook [14] and the Centre for Reviews and Dissemination [15]. The TLR was performed in the Ovid MEDLINE^®^ In-Process & Other Non-Indexed Citations and Ovid MEDLINE^®^ databases, with the search for articles published from January 2012 through September 2022 (except for RCTs, for which no time restriction was applied) focusing on the most recent data. The gray literature search was conducted in January 2023 and included targeted, iterative manual searches of 29 regulatory and/or research organization websites, which are listed in Supplementary Table 1.

### Eligibility criteria

Abstracts and full texts were screened by a reviewer who selected relevant articles based on the eligibility criteria (Supplementary Table 2). The eligibility criteria included studies focusing on adult patients diagnosed with HF (NYHA II-IV) and LVEF ≥ 40% (symptomatic HFmrEF/HFpEF). The analysis considered various outcomes, including epidemiologic data, clinical and humanistic burden, treatment overviews, economic burden, and economic evaluations. To ensure the comprehensive coverage of evidence, the study design criteria included real-world evidence (RWE), RCTs, treatment guidelines, systematic literature reviews (SLRs), cost-effectiveness analyses (CEAs), and budget impact analyses (BIAs). There were no restrictions regarding the study design for economic burden studies. Studies eligible for inclusion had to be published in English, except for treatment guidelines, which were considered irrespective of language.

A process of study prioritization was then undertaken to identify those articles most likely to provide valuable insights into the research topics. During the prioritization process, studies conducted in populations of patients with acute HF were excluded. Furthermore, those that used medical devices, diagnostic tests, dietary supplements, and genetic testing with regard to behavioral interventions were excluded. Pilot studies, phase 1 and phase 2 trials, pooled analyses, reviews, and SLRs were also excluded. Additionally, studies were excluded if their sample size was < 50 patients for RCTs or < 100 patients for RWE studies. For RCTs, only studies evaluating the most commonly used and recommended medications according to the guidelines [SGLT-2is, sacubitril/valsartan, mineralocorticoid antagonists (MRAs), angiotensin-converting enzyme inhibitors (ACE-Is), and angiotensin receptor blockers (ARBs)] were included. Moreover, only the latest guidelines were considered. For RWE studies reporting epidemiology, comorbidities, effectiveness, safety, treatment patterns, or economic burden, only studies with data collection end dates in ≥ 2016 were included.

### Data extraction

The epidemiologic outcomes of interest included the incidence, prevalence, mortality, and co-morbidities related to HFpEF and/or HFmrEF. Management outcomes of interest included the treatment pattern/practice, percentage of patients receiving each treatment type, adherence/compliance, discontinuation rate and adverse events, predictors, and risk factors for HF. To assess clinical burden, the following outcomes were collected: CV death; HF events; improvement in NYHA class; non-fatal CV events; composite renal events [defined as a sustained decrease in estimated glomerular filtration rate (eGFR) ≥ 50%, a sustained decrease in eGFR ≥ 57%, a sustained eGFR decline to < 15 ml/min/1.73m^2^, and the initiation of dialysis or renal transplantation]; changes in UACR from baseline; new onset of atrial fibrillation; and hospitalizations (all-cause and CV). The humanistic burden outcome of interest was health-related quality of life (HRQoL). Economic burden outcomes included direct costs, indirect costs, and resource use.

### Quality assessment and risk of bias

The quality of the included RCTs was assessed using the Cochrane Risk of Bias Tool checklist [14]. For the included cohort studies, their methodologic quality was assessed using the Newcastle–Ottawa Scale (NOS) or an adapted version of the NOS in the case of cross-sectional studies [16].

## Results

### Literature search results

**Fig. 1:**
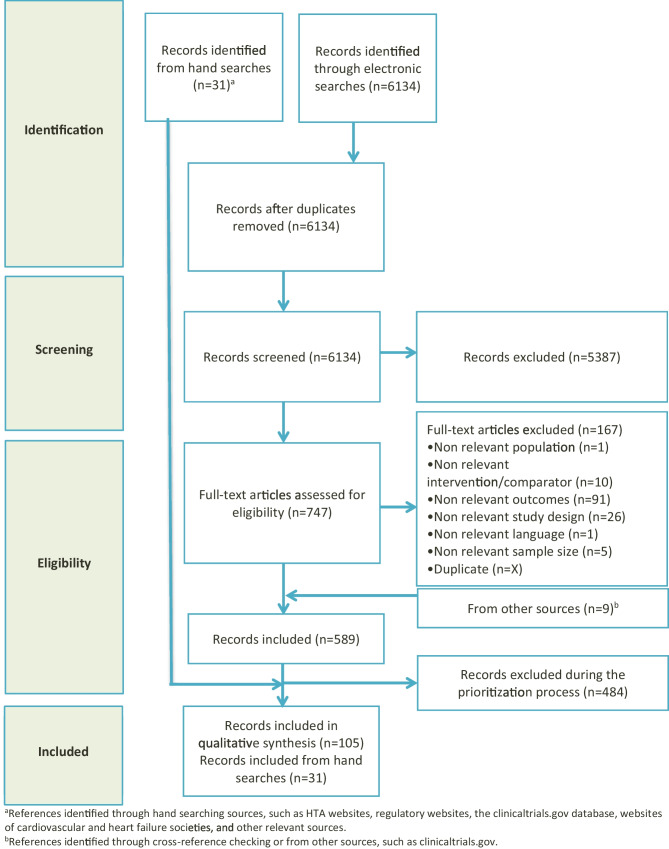
Preferred Reporting Items for Systematic Reviews and Meta-Analysis (PRISMA) flow diagram

**Fig. 2:**
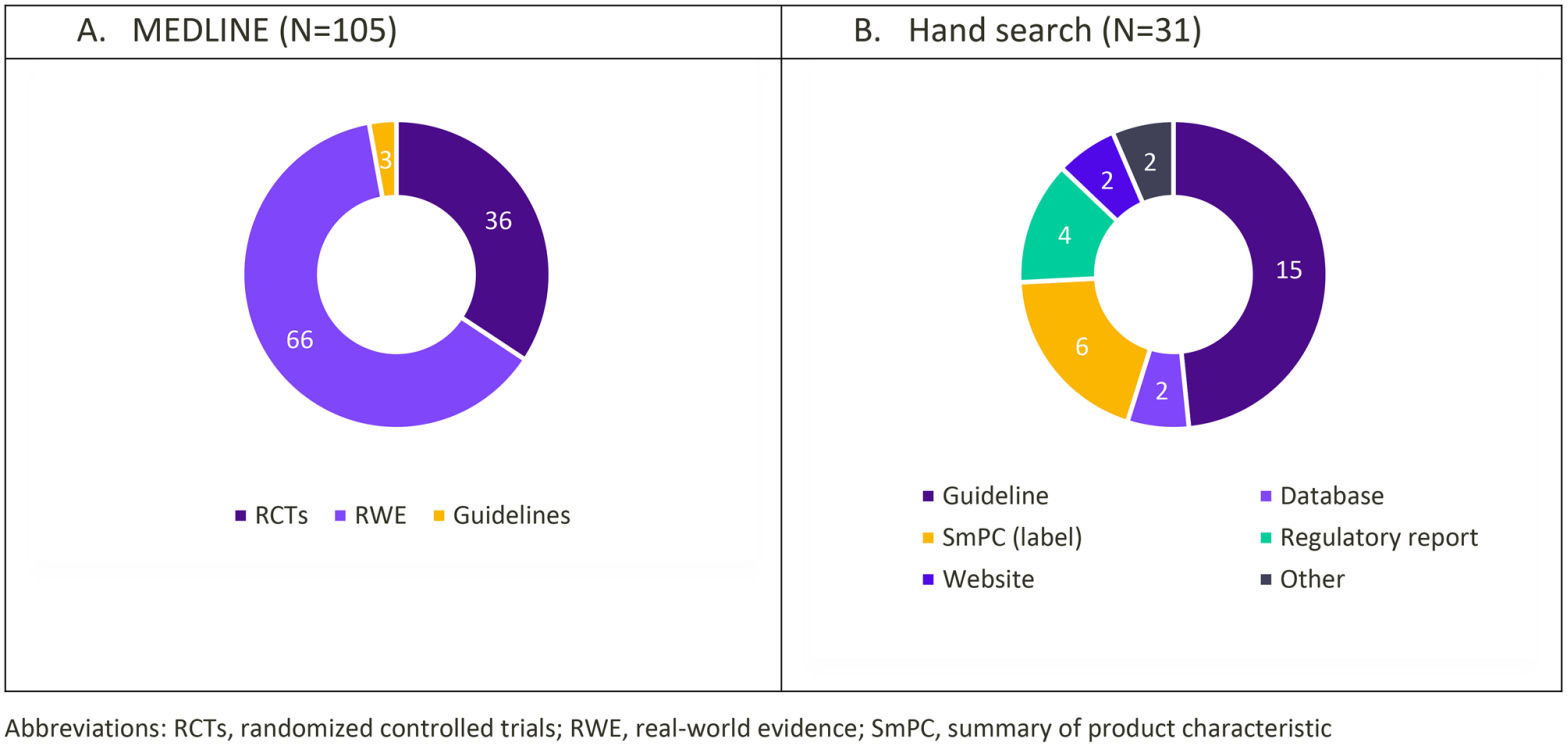
Distribution of included studies by study design (**A**) or type of document (**B**)

The electronic searches yielded 6134 records after the de-duplication process. A total of 580 records met the relevant criteria. An additional 9 records were obtained from cross-reference checking or from other sources, such as clinicaltrials.gov. Following the prioritization process, 105 records were included in the qualitative synthesis. Moreover, 31 records were sourced from manual searches. The study selection process is depicted in Fig. 1. Additionally, the distribution of included studies by study design and type of document is presented in Fig. 2. The characteristics of the RCTs and RWE included in the TLR are presented in Tables 1 and 2. The review found 18 guidelines for HF (including HFmrEF/HFpEF) from 6 countries (the United States, Japan, France, Germany, Sweden, and the United Kingdom) published between 2014 and 2022. Supplementary Table 3 provides an overview of these guidelines and their recommendations for HFmrEF/HFpEF care.

**Table 1:** Characteristics of randomized controlled trials included in the targeted literature review

**Study ID**	**Study design; follow-up**	**Country**	**Population**	**N**	**Interventions**	**Composite outcome**^**b**^	**CV death**	**HF events**	**Hospitalization (all-cause, CV)**	**Non-fatal CV event**	**UACR change from baseline**	**Composite renal outcome**	**Safety**	**HRQoL/PROs**
HFpEF-specific trials (including HFmrEF)														
Aldo-DHF [131]	RCT (1:1; phase: NR), DB; mean 11.6 m	Multinational (Europe)	Adults with HF (NYHA II-III) and LVEF ≥ 50%	422	Spironolactone Placebo	NR	NR	NR	✓	NR	NR	NR	✓	✓
CANONICAL [132]	RCT (1:1; phase: NR), parallel, OL; 6 m	Japan	Adult patients with HF (NYHA II-III) and LVEF ≥ 50% with T2D	82	Canagliflozin Standard diabetic therapy	NR	✓	✓	NR	NR	NR	NR	✓	NR
CHARM-Preserved [83]	RCT (1:1; phase III), parallel, DB; median 36.6 m	Multinational (global)	Adult patients with CHF (NYHA II-IV) and LVEF > 40%	3023	Candesartan Placebo	✓ ^1, 2^	✓	✓	NR	✓	NR	NR	✓	NR
DELIVER [79]	RCT (1:1; phase III), parallel, DB; median 27.6 m	Multinational (global)	Adult patients with HF (NYHA II-IV) and LVEF > 40%	6263	Dapagliflozin Placebo	✓ ^1^	✓	✓	NR	NR	NR	NR	✓	✓
DETERMINE-Preserved [133]	RCT (1:1; phase III), parallel, DB; 4 m	Multinational (global)	Adult patients with HF (NYHA II-IV) and LVEF > 40%	504	Dapagliflozin Placebo	NR	NR	NR	NR	NR	NR	NR	✓	✓
EMPERIAL-Preserved [71]	RCT (1:1; phase III), parallel, DB; NR	Multinational (global)	Adult patients with HF (NYHA II-IV) and LVEF > 40%	315	Empagliflozin Placebo	NR	NR	NR	NR	NR	NR	NR	✓	✓
EMPEROR-Preserved [82]	RCT (1:1; phase III), parallel, DB; median 26.2 m	Multinational (global)	Adults with CHF (NYHA II-IV) and LVEF > 40%	5988	Empagliflozin Placebo	✓ ^1^	✓	✓	✓	NR	NR	✓	✓	✓
Feng 2022 [35]	RCT (1:1; phase: NR), parallel, Blinding: NR; NR	China	Adult patients with HFpEF (NYHA II-III) and LVEF ≥ 40%	78	Sacubitril/valsartan Basic treatment^a^	✓ ^3^	NR	✓	NR	NR	NR	NR	✓	✓
I-PRESERVE [134]	RCT (1:1; phase III), parallel, DB; mean 49.5 m	Multinational (global)	Adult patients with HF (NYHA II-IV) and LVEF ≥ 45%	4128	Irbesartan Placebo	✓ ^5, 6, 7, 8^	✓	✓	✓	NR	NR	NR	✓	✓
PARAGON-HF [85]	RCT (1:1; phase III), parallel, DB; median 35 m	Multinational (global)	Patients with CHF (NYHA II-IV) and LVEF ≥ 45%	4822	Sacubitril/valsartan Valsartan	✓^1^	✓	✓	NR	NR	NR	✓	✓	✓
PARALLAX [32]	RCT (1:1; phase III), parallel, DB; 6 m	Multinational (global)	Adults with CHF (NYHA II-IV) and LVEF > 40%	2556	Sacubitril/valsartan Background medication-based individualized comparator	NR	NR	NR	NR	NR	NR	NR	✓	✓
PEP-CHF [84]	RCT (1:1; phase: NR), parallel, DB; mean 26.2 m	Multinational (global)	Elderly patients (> 70 years old) with HF, LVEF > 40% and treated with diuretics	850	Perindopril Placebo	✓ ^6^	✓	✓	NR	NR	NR	NR	✓	NR
PRESERVED-HF [80]	RCT (1:1; phase IV), parallel, DB; 3.25 m	US	Adult patients with HF (NYHA II-IV) and LVEF ≥ 45%	324	Dapagliflozin Placebo	NR	NR	✓	NR	NR	NR	NR	✓	✓
STRUCTURE [135]	RCT (1:1; phase IV), parallel, SB; 6 m	Multinational (Europe)	Adult patients with HF (NYHA II-III) and LVEF > 50%	150	Spironolactone Placebo	NR	NR	NR	✓	NR	NR	NR	✓	NR
TOPCAT [86]	RCT (1:1; phase III), parallel, DB; mean 39.6 m	Multinational (global)	Adult patients with symptomatic HF and LVEF ≥ 45%	3445	Spironolactone Placebo	✓^4^	✓	✓	✓	✓	NR	NR	✓	✓
Upadhya 2017 [136]	RCT (1:1; phase: NR), parallel, DB; 9 m	US	Elderly patients with HF (NHANES ≥ 3) and LVEF ≥ 50%	80	Spironolactone Placebo	NR	NR	NR	✓	NR	NR	NR	✓	✓
Yip 2008 [137]	RCT (1:1:1; phase: NR), parallel, OL; 12 m	Multinational (Asia)	Adult patients with HF (NYHA II-III) and LVEF > 45%	150	Diuretics plus irbesartan/diuretics plus ramipril Diuretics alone	NR	✓	✓	NR	NR	NR	NR	✓	✓
Yuksek 2019 [138]	RCT (1:1; phase: NR), parallel, Blinding: NR; 11 m	Multinational (Asia/Europe)	Adult patients with symptomatic DHF and LVEF ≥ 50%	108	Perindopril Standard DHF treatment	NR	NR	NR	NR	NR	NR	NR	✓	NR
Zi 2003[139]	RCT (1:1; phase: NR), parallel, Blinding: NR; NR	Multinational (Europe)	Elderly patients with HF (NYHA II-III) and LVEF ≥ 40%	74	Quinapril Placebo	NR	NR	✓	NR	NR	NR	NR	✓	✓
Non HFpEF-specific trials														
ALLHAT [140]	RCT (1.7:1:1:1^c^; phase III), parallel, DB; 96 m	NR	Adult patients with HF, LVEF ≥ 50%, and hypertension	1367 (HFpEF: 404)	Chlorthalidone Lisinopril	NR	NR	NR	✓	NR	NR	NR	NR	NR
CANDLE [141]	RCT (1:1; phase: NR), parallel, OL; 6 m	Japan	Adult patients with T2D & CHF (NYHA I to III) and LVEF ≥ 50%	233 (HFpEF: 165)	Canagliflozin Glimepiride	NR	NR	NR	NR	NR	NR	NR	NR	✓
CHIEF-HF [142]	RCT (1:1; phase III), parallel, DB; 6 m	US	HF patients aged ≥ 18 years	476 (HFpEF: 267)	Canagliflozin Placebo	NR	NR	NR	NR	NR	NR	NR	NR	✓
DECLARE-TIMI [53]	RCT (1:1; phase III), parallel, DB; median 50.4 m	Multinational (global)	Adult patients with HF, T2D, and LVEF ≥ 45%	17,160 (with documented HFmrEF/HFpEF: 808)	Dapagliflozin Placebo	✓ ^1^	✓	✓	NR	NR	NR	✓	✓	NR
SCORED [34]	RCT (1:1; phase III), parallel, DB; median 16.0 m (intervention) and 15.9 m (placebo)	Multinational (global)	Adult patients with T2D, CKD, risk of CV disease, and LVEF ≥ 40	10,584 (HFpEF: 1667; HFmrEF: 581)	Sotagliflozin Placebo	✓ ^1^	NR	NR	NR	NR	NR	NR	NR	NR
SOLOIST-WHF [34]	RCT (1:1; phase III), parallel, DB; median 9 m	Multinational (global)	Patients aged < 18 years or > 85 years with T2DM	1222 (HFpEF: 256)	Sotagliflozin Placebo	✓ ^1^	NR	NR	NR	NR	NR	NR	NR	NR
SUPPORT [55]	RCT (1:1; phase III), parallel, OL; median 52.8 m	Japan	Adult patients with chronic HF (NYHA II-IV) and hypertension	1147 (HFpEF: 709; HFmrEF: 213)	Olmesartan Control group^d^	NR	NR	NR	NR	NR	✓	NR	✓	NR
VERTIS CV [52]	RCT (1:1:1; phase III), parallel, DB; median 42 m	Multinational (global)	Adult patients with T2D, HF, and LVEF ≤ 45%	8246 (HFpEF: 1007)	Ertugliflozin Placebo	✓ ^1^	✓	✓	NR	NR	NR	NR	NR	NR

*CHF* chronic heart failure, *CV* cardiovascular, *DB* double-blinded, *DHF* diastolic heart failure, *HF* heart failure with preserved ejection fraction, *HFmrEF* heart failure with mildly reduced ejection fraction, *HHF* hospitalization due to heart failure, *HRQoL* health-related quality of life, *LVEF* left ventricular ejection fraction, *MI* myocardial infarction, *m* month(s), *N* number, *NR* not reported, *NYHA* New York Heart Association, *OL* open-label, *PROs* patient-reported outcomes, *RCT* randomized controlled trial, *T2D* type 2 diabetes, y year(s)

✓ data available

^a^Diovan^®^, β-blockers, calcium channel blockers, nitrate drugs, and antiplatelet drugs

^b^Composite outcome: 1. CV death or HHF; 2. CV death, HHF or CV events; 3. CV death or CV events; 4. CV death or HHF or aborted cardiac arrest; 5. all-cause death or hospitalization for CV; 6. all-cause death or HHF; 7. CV, MI, or stroke death; 8. HF death or HHF

^c^Participants were randomly assigned to step 1 drugs—chlorthalidone, amlodipine, lisinopril, or doxazosin—at a ratio of 1.7:1:1:1

^d^Renin-angiotensin system inhibitors and/or β-blockers

**Table 2:** Characteristics of real-world evidence included in the targeted literature review

Study ID	Study type; follow-up	Country	Population	Sample size	Interventions	Incidence	Prevalence	Mortality	Comorbidities	Treatment patterns	Effectiveness^a^	Safety	HRQoL/PROs	Economic burden
Europe														
Cohen Solal, 2022 [60] (CARNEFER)	Prospective, cross-sectional study; NR	France	Patients with HF	1661 (HFpEF: 527)	NA	NR	✓	NR	✓	✓	NR	NR	NR	NR
Fayol, 2022 [74]	Retrospective, cohort study; 2.17 y	France	Adult patients hospitalized for congestive HF	2180 (HFpEF: 928)	NA	NR	✓	✓	✓	NR	✓ ^6^	NR	NR	NR
Tamisier, 2020 [143] (FACE)	Prospective, cohort study; 24 m	France	Adult patients with CHF	503 (HFpEF: 233)	ASV + SoC/SoC	NR	✓	NR	NR	NR	NR	NR	✓	NR
Gobel, 2021 [66] (MyoVasc)	Prospective, cohort study; 72 m	Germany	Adult patients suffering from systolic or diastolic cardiac dysfunction or HF	3289 (HFpEF: 646)	NA	NR	✓	NR	✓^a^	✓^a^	NR	NR	NR	NR
Wenzel, 2022 [47] (HCHS)	Prospective, cohort study; 48 m	Germany	Adult patients from the HCHS with available echocardiography data	7074 (HFpEF: 155)	NA	NR	✓	NR	✓	✓	NR	NR	NR	NR
Garg, 2021 [59] (SHEAF)	Prospective, cohort study; NR	UK	Patients with suspected HF	6272 (HFpEF: 2022)	NA	NR	✓	✓	✓	NR	✓^6^	NR	NR	NR
Hawley, 2020 [144]	Retrospective, cohort study; two 12 m-periods separated by 12 m	UK	Adult patients with HF admitted over two 12-month periods before (2012/2013) and after (2015/2016) IHFS establishment	Period 1 (2012–2013): 350^b^ Period 2 (2015–2016): 505^b^ (HFpEF: NR)	NA	NR	NR	✓	NR	NR	✓^3a,4a^	NR	NR	NR
Bekfani, 2019 [45] (SICA-HF)	Prospective, cross-sectional study; NR	Germany, England, Slovenia	Outpatients with HF	190	NA	NR	NR	NR	NR	NR	NR	NR	✓	NR
Meyer, 2021 (SwedeHF) [44]	Registry study; median FU: 2 y	Sweden	Patients with HFpEF	14,434	Beta-blocker No beta-blocker	NR	NR	✓	NR	✓	✓^2, 3c, 4a^	NR	NR	NR
Lindberg, 2022 [61]	Prospective, registry study; 30 m	Sweden	Adult patients with available data on EF	75,518 (HFpEF: 18,225)	NA	NR	✓	✓	✓	✓	✓^2, 3b^	NR	NR	NR
Savarese, 2021 [13] (SwedeHF)	Registry study; NR	Sweden	Patients with HF	114,737 (HFpEF: 3710)	NA	NR	NR	✓	NR	✓	✓ ^1a, 2, 3b^	NR	NR	NR
Streng, 2018 [99] (BIOSTAT-CHF)	Retrospective, cohort study; 25 m	Multinational (Europe)	Patients with HF	3499 (HFpEF: 556; HfmrEF: 634)		NR	NR	NR	✓	NR	NR	NR	✓	NR
Uijl, 2021 [36] (SwedeHF)	Registry study; NR	Sweden, Netherlands	Patients with HF	9062	NA	NR	NR	✓	✓	✓	✓^1a, 2, 3c^	NR	NR	NR
Verdu-Rotellar, 2020 [145] (HEFESTOS)	Prospective, cohort study; 1 m	Spain, France, Ireland, Germany, Italy, Slovenia, Croatia, Bulgaria, Hungary, Sweden	Adult patients with HF who attended primary care centers, or who were managed by primary care physicians via home care, because of HF decompensation	692 (HFpEF: NR)	NA	NR	NR	NR	✓^a^	✓^a^	✓^a,1c, 6^	NR	NR	NR
**US**														
Afzal, 2022 [87]	Retrospective, registry study; 156 m	US	Adult patients with HF	6,403,626^b^ (HFpEF: 2,545,286)^b^	NA	NR	NR	✓	NR	NR	✓ ^3c^	NR	NR	✓
Ambrosy, 2021 [90] (UTILISE-WHF)	Retrospective, cohort study; NR	US	Adult patients hospitalized for WHF	118,002 pts; 287,992^b^ (HFpEF: 142,347)	NA	NR	✓	NR	NR	NR	✓ ^3c^	NR	NR	NR
Arnold, 2018 [77]	Retrospective, registry study; NR	US	Adult patients with HF	456,106 (HFpEF: 54,542)	NA	NR	✓	NR	✓^a^	✓	NR	NR	NR	NR
Arora, 2020 [146]	Retrospective, cohort study; 12 m	US	Patients with AF and comorbid HF	119,694 (HfpEF: 56,395)	NA	NR	NR	✓	✓	NR	✓ ^1c, 3a, 4a, 4b^	NR	NR	✓
Brann, 2020 [49]	Retrospective, cohort study; 26.88 m	US	Adult patients with HF	448	NA	NR	NR	NR	✓	✓	NR	NR	NR	NR
Buckallew, 2021 [147]	Retrospective, cohort study; NR	US	Adult patients with HF and CKD (stages 3–5)	121 (HFpEF: 63)	Spironolactone	NR	NR	NR	NR	✓^a^	NR	NR	NR	NR
Clark, 2022 [88]	Retrospective, cohort study; 120 m	US	Adult patients with admission diagnoses of HF	11,693,994^b^ (HFpEF: 3,605,004)^b^	NA	NR	NR	✓	NR	NR	✓ ^3c^	NR	NR	✓
Contreras, 2019 [148] (PINNACLE)	Retrospective, cohort study; NR	US	Patients with non-valvular AF and congestive HF	340,127 (HFpEF: 210,917)	NA	NR	NR	NR	✓^a^	NR	NR	NR	✓	NR
Davies, 2022 [48]	Retrospective, cohort study; 87 m	US	Patients with HF	1018	NA	NR	NR	NR	✓	NR	NR	NR	NR	NR
Desai, 2021 [91]	Cohort study; 53 m	US	Adult patients with HF (≥ 65 y)	3,134,414 (HFpEF: 2,933,464)	NA	NR	✓	✓	✓	✓	✓^1d, 2^	NR	NR	NR
Desai, 2022 [92]	Prospective, cohort study; FU > 229,824 PY	US	Adult patients with HFpEF	621,171	Spironolactone	NR	NR	NR	✓	✓	NR	✓	NR	NR
Dunlay, 2021[149]	Retrospective, cohort study; NR	US	Patients with advanced HF	6836 (HFpEF: 406)	NA	NR	✓	NR	✓^a^	NR	✓^a 3e, 4a, 6^	NR	NR	NR
Fudim, 2020 [43] (GWTG-HF)	Retrospective, registry study; NR	US	Patients with HFpEF	142,201	MRAs/No MRAs	NR	NR	NR	✓	✓	NR	NR	NR	✓
Greenberg, 2019 [72]	Retrospective, cohort study; median: 12.2 m	US	Adult patients with ≥ 1 HF diagnosis during hospitalization or an emergency room visit	7005 (HFpEF: 4288)	NA	NR	NR	✓	✓	✓	✓ ^6^	NR	NR	✓
Hamo, 2021 [150] (GWTG-HF)	Prospective, registry study; NR	US	Patients with HF	335,735 (HFpEF: 142,595)	NA	NR	✓	✓	NR	NR	✓^6^	NR	NR	NR
Harmon, 2020 [37]	Retrospective study; NR	US	Adult patients with HF	492	NA	NR	NR	NR	✓	✓	✓^3a^	NR	NR	NR
Hornsby, 2019 [42] (CHIP)	Prospective, cohort study; ≥ 6 m	US	Adult patients with HF	114	NA	NR	NR	✓	✓	NR	✓ ^1e, 3c, 4a, 4b^	NR	NR	NR
Ibrahim, 2019 [65] (PINNACLE)	Prospective, registry study; NR	US	Adult patients with HF	697,542 (HFpEF: 324,387)	NA	NR	✓	NR	✓	✓	NR	NR	NR	NR
Jentzer, 2022 [73]	Retrospective, cohort study; NR	US	Adult patients with HF admitted to CICU	4012 (HFpEF: 1293)	NA	NR	✓	✓	✓	NR	✓ ^4a^	NR	NR	✓
Joseph, 2013 [96]	Prospective, registry study; median FU: 16.6 m	US	Patients with HF	849 (HFpEF: 200)	NA	NR	NR	NR	✓^a^	NR	✓^1e, 4a^	NR	✓	NR
Joyce, 2016 [151]	Retrospective, cross-sectional study; NR	US	Adult patients with ambulatory HF	726 (HFpEF: 115)	NA	NR	NR	NR	✓^a^	✓^a^	NR	NR	✓	NR
Lam, 2021 [100]	Retrospective, cohort study; Median FU: 18 m (HFpEF: 20 m)	US	Patients with HF	109,721 (HFpEF: 33,781)	NA	NR	NR	NR	✓	✓	✓ ^4a^	NR	NR	✓
Li, 2019 [50]	Retrospective, cohort study; 54 m	US	Adult patients with HF	1852	NA	NR	NR	NR	✓	✓	NR	NR	NR	NR
Lopuszynski, 2021 [152]	Retrospective, cohort study; median FU: 21.7 m	US	Patients with HFpEF	487	NA	NR	NR	✓	✓	NR	✓^6^	NR	NR	NR
Lorenzo, 2022 [62]	Retrospective, cohort study; mean FU: 208.6 days	US	Adult patients with HF	874 (HFpEF: 531)	NA	NR	✓	NR	✓	✓	NR	NR	NR	NR
Luo, 2018 [153] (GWTG-AFIB)	Prospective, registry study; NR	US	Patients with HF	10,883 (HFpEF: 5516)	NA	NR	✓	NR	✓^a^	✓	NR	NR	NR	NR
Murtaza, 2020 [120]	Retrospective, cohort study; 46 m	US	Adult patients with HF, > 3 m FU, and interpretable Doppler echocardiograms	633 (HFpEF: 269)	NA	NR	NR	✓	✓	✓	✓ ^3a^	NR	NR	✓
Navid, 2021 [154]	Retrospective, cohort study; NR	US	Patients with HFpEF	134	NA	NR	NR	NR	✓	✓	NR	NR	NR	NR
Patel, 2021 [155] (GWTG-HF)	Prospective, registry study; NR	US	Adult patients with HF	365,494 (HFpEF: 159,702)^b^	NA	NR	✓	✓	NR	NR	✓^6^	NR	NR	NR
Perry, 2022 [46]	Retrospective, cohort study; 37.2 m	US	Adult patients with HF with an improvement in LVEF from < 40% to ≥ 53%	133	NA	NR	NR	NR	✓	✓	NR	NR	NR	NR
Regmi, 2020 [156]	Retrospective, cohort study; NR	US	Adult patients discharged from hospital service	1781 (HFpEF: 200)	NA	NR	NR	NR	NR	NR	✓ ^4a^	NR	NR	NR
Reinhardt, 2021 [89]	Retrospective, cohort study; NR	US	Adult patients with HF	10,392,189^b^ (HFpEF: 3,117,059)^b^	NA	NR	NR	NR	NR	NR	✓^4a^	NR	NR	✓
Subramaniam, 2022 [57]	Retrospective, cohort study; NR	US	Adult patients with incident HF	4597 (HFpEF: 2438)	NA	✓	NR	NR	✓^a^	NR	NR	NR	NR	✓^a^
Wohlfahrt, 2021 [111]	Prospective, cohort study; 12 m	US	Adult patients with HF who completed PRO assessments	319 (HFpEF: 107)	NA	NR	NR	NR	✓	✓	NR	NR	✓	NR
Yee, 2019 [157]	Prospective, cohort study; 24 m	US	Patients with HF	738 (HFpEF: 151)	NA	NR	NR	✓	✓	NR	✓ ^1f, 4a^	NR	NR	NR
Japan														
Aizawa, 2022 [67]	Retrospective, registry study; median FU: 19 m	Japan	Adult patients with HF undergoing maintenance hemodialysis	142 (HFpEF: 63)	NA	NR	✓	NR	✓^a^	NR	NR	NR	NR	NR
Kinugawa, 2019 [158] (SMILE)	Prospective, cohort study; NR	Japan	Adult patients with HF accompanied by fluid retention who received tolvaptan	1741 (HFpEF: 795)	TLV	NR	✓	NR	✓	✓	✓^3d^	✓	NR	NR
Kiuchi, 2019 [101]	Retrospective, cohort study; NR	Japan	Adult patients who were hospitalized with HF and initiated tolvaptan	204 (HFpEF: 108)	TLV	NR	✓	NR	NR	✓	NR	NR	NR	✓
Seki, 2022 [76]	Retrospective, cohort study; 31 m	Japan	Patients with congestive HF taking tolvaptan	147 (HFpEF: 77)	TLV	NR	✓	✓	✓	✓	✓^2^	NR	NR	NR
Shiga, 2019 [63]	Retrospective, cohort study; median FU: 19 m	Japan	Hospitalized patients with decompensated HF	1245 (HFpEF: 538)	NA	NR	✓	✓	✓	✓	✓^2, 4a^	NR	✓	✓
Suzuki, 2019 [159]	Prospective, cohort study; 14.96 m	Japan	Adult patients with HF	155 (HFpEF: 64)	HF treatment	NR	✓	✓	✓	✓	✓ ^1 g, 3c, 4b, 5^	✓	NR	NR
Takahari, 2019 [40]	Retrospective, cross-sectional study; NR	Japan	Adult patients who underwent ESE and CPET	139	NA	NR	NR	NR	NR	✓	NR	NR	NR	NR
Tomii, 2021 [160]	Retrospective, cohort study; NR	Japan	Patients with HF	330 (HFpEF: 270)	NA	NR	✓	NR	✓^a^	NR	✓^a, 3c, 6^	NR	NR	NR
Tsukamoto, 2021 [68]	Prospective, cohort study; 12 m	Japan	Hospitalized patients with decompensated HF	1410 (HFpEF: 522)	NA	NR	✓	✓	✓	✓	✓^2^	NR	NR	NR
Yoshihisa, 2019 [69]	Prospective, cohort study; median FU: 40 m	Japan	Patients with decompensated HF who were discharged from hospital	2103 (HFpEF: 1161)	NA	NR	✓	NR	✓	✓	✓^a, 1a, 2, 4c, 6^	NR	NR	✓^a^
Yoshihisa, 2020 [41]	Prospective, cohort study; 6 m	Japan	Adult patients with HFpEF at first LVEF assessment	1082	NA	NR	NR	NR	✓	✓	✓^3f^	NR	NR	NR
International														
Chandramouli, 2019 [161] (ASIAN-HF)	Prospective, registry study; NR	Taiwan, Hong Kong, China, India, Malaysia, Thailand, Singapore, Indonesia, the Philippines, Japan, and South Korea	Patients with HF	5964 (HFpEF: 139)	NA	NR	✓	NR	NR	NR	NR	NR	NR	NR
Tromp, 2018 [70] (ASIAN-HF)	Prospective, registry study; NR	Taiwan, Hong Kong, China, India, Malaysia, Thailand, Singapore, Indonesia, the Philippines, Japan, and South Korea	Adult patients with HF	6480 (HFpEF: 1204)	NA	NR	✓	NR	✓^a^	NR	✓^a, 1a, 4c, 6^	NR	NR	NR
Tromp, 2019 [56] (Asian-HF)	Prospective, registry study; NR	Taiwan, Hong Kong, China, India, Malaysia, Thailand, Singapore, Indonesia, the Philippines, Japan, and South Korea	Adult patients with HF and DM	6438 (HFpEF: 561)	NA	NR	✓	NR	NR	NR	NR	NR	✓^a^	NR
Hage, 2020 [38] (PROMIS-HFpEF)	Prospective, cohort study; 12 m	NR	Patients with chronic but stable HFpEF undergoing CFR measurements	257	NA	NR	NR	NR	✓	✓	✓^1a, 1b, 2, 3a, 3b, 4a^	NR	✓	NR
Kapelios, 2020 [162] (ESC-LTR)	Prospective, registry study; NR	Austria, Bosnia and Herzegovina, Bulgaria, Czech Republic, France, Egypt, Greece, Hungary, Israel, Italy, Lithuania, Poland, Latvia, Portugal, Romania, Serbia, Slovakia, Slovenia, Spain, Sweden, and Turkey	Patients with HF	8130 (HFpEF: 1502)	NA	NR	NR	NR	NR	NR	✓^a, 1f, 4c 6^	NR	NR	NR
Shah, 2018 [39] (PROMIS-HFpEF)	Prospective, cohort study; NR	Sweden, Finland, the US, and Singapore	Adult patients with a confirmed diagnosis of CHF	202	NA	NR	NR	NR	✓	✓	✓ ^1a, 1b, 2, 4a, 4b^	NR	✓	NR

*AF *atrial fibrillation, *CFR* coronary flow reserve, *CHF* chronic heart failure, *CICU* cardiac intensive care unit *CKD* chronic kidney disease *CV* cardiovascular, *DM* diabetes mellitus, *FU*, follow-up, *HF* heart failure, *HFmrEF* heart failure with midrange ejection fraction, *HFpEF* heart failure with preserved left ventricular ejection fraction, *HRQoL* health-related quality of life, *LVEF* left ventricular ejection fraction, *MI* myocardial infarction, *NR* not reported, *NYHA* New York Heart Association, *PRO* patient-reported outcomes, *PY* patient-years, *RWE* real-world evidence, *TLV* tolvaptan, *UK* United Kingdom, *US* United States, *WHF* worsening heart failure

^a^Effectiveness: 1. Composite outcome (1a, CV death or hospitalization for HF; 1b, all-cause death or hospitalization for HF [including first and recurrent]; 1c, all-cause death or HF readmission; 1d, all-cause death or worsening HF; 1e, death or hospitalization; 1f, death or other events [i.e., transplant or ventricular assist device implant]; 1 g, composite of cardiac events [all-cause death, non-fatal MI, non-fatal stroke, and hospitalization for HF]); 2. CV death; 3. HF events (3a, readmissions for HF; 3b, first hospitalization for HF; 3c, hospitalization for HF [overall]; 3d, worsening HF; 3e, progression to advanced HF; 3f, worsened LVEF); 4. hospitalizations (4a, all-cause hospitalizations; 4b, CV hospitalizations; 4c, hospitalizations for HF); 5. non-fatal CV events; 6. all-cause death

^b^Data for HFpEF and/or HFmrEF not available; only data for an overall HF population were provided

^c^Sample size provided as the number of hospitalizations/admissions

### Definition and diagnosis

The general definition of chronic HF was specified in 12 out of 18 clinical guidelines [1–3, 17–25]. HFpEF was defined in 15 [1–3, 17–20, 23–30] of 18 [1–3, 17–31] guidelines; HFmrEF was defined in 10 of 18 guidelines [1–3, 17, 19, 20, 23, 24, 27, 28]. The HFmrEF/HFpEF population is often grouped together, but they are well defined in the latest heart failure clinical guidelines for the United States, Europe, and Japan based on their LVEF values [1–3]. The guidelines set an LVEF cut-off of ≥ 50% for HFpEF and between 40 to 41% and 49% for HFmrEF. Additionally, the guidelines describe more subgroups in the HFmrEF/HFpEF population, including patients transitioning between LVEF categories, which may present different outcomes, such as HF with improved EF (patients whose LVEF improved from < 40% to > 40%) [1–3]. However, there were variations in the definition of HFpEF in the included RCT and RWE studies compared with the guidelines, represented by differences in LVEF thresholds (> 40%, ≥ 45%, or ≥ 50%). HFmrEF, usually considered part of HFpEF, was not explicitly defined in these studies, with most of them using an LVEF cut-off of > 40% or ≥ 45%. RCTs usually include subgroup analyses in the population of patients with LVEF < 50% (corresponding to the HFmrEF population, per the guidelines) [13, 32–55]. HFpEF and HFmrEF are 2 groups of HF characterized by a complex pathophysiology and overlapping symptoms, making their diagnoses challenging. Multiple risk factors and causes contribute to these conditions, and their phenotypic manifestations can vary [2, 26]. Despite ongoing HF research, information from the literature on the specific predictors and risk factors for the HFmrEF/HFpEF population is scarce. Only 2 included studies reported limited information on the predictors and risk factors for HFpEF and advanced HF [56, 57]. One study indicated a higher likelihood of HFpEF in participants with diabetes and microvascular complications [56], aligning with calls for further research on the disease’s pathophysiology and natural history made in the literature [58]. Diagnosing HFmrEF/HFpEF is challenging because of its nonspecific signs and symptoms, which can overlap with other conditions [2]. Therefore, cardiac imaging and the measurement of natriuretic peptides (NPs) play a crucial role in diagnosis. Guidelines propose specific diagnostic criteria, with an NT proBNP value threshold > 125 pg/ml commonly used for HFpEF diagnosis. However, challenges remain and different guidelines recommend various diagnostic algorithms, like H_2_FPEF (heavy, 2 or more hypertensive drugs, atrial fibrillation, pulmonary hypertension, elder age > 60, elevated filling pressures) or HFA-PEFF (Heart Failure Association-pre-test assessment, echocardiography and natriuretic peptide score, functional testing, final aetiology) scores [1–3, 17, 26], leading to different patient classifications [1, 2]. Limited access to specialized tests may hinder the practicality of these scores, contributing to ongoing diagnostic uncertainty in HFpEF [1]. To address this, a simplified pragmatic approach was recommended by the European Society of Cardiology (ESC) 2021, German Cardiac Society 2021, and US 2022 guidelines, focusing on widely available variables for diagnosing HFpEF (Table 3) [1, 2, 17]. The generalizability of the scores used for HFpEF diagnosis has been tested in various trials and cohorts, resulting in a varying diagnostic performance [1]. In a few guidelines, the HFmrEF diagnostic criteria align with HFpEF [1, 2, 17, 23, 28]. The diagnosis of HFmrEF requires the presence of symptoms and/or signs of cHF, and a mildly reduced EF (LVEF measurement). The presence of elevated NPs and other evidence of structural heart disease make the diagnosis more likely but are not mandatory for diagnosis if there is certainty regarding the measurement of LVEF [1]. The main criteria used in RCTs is similar to those mentioned in clinical guidelines, relying on symptoms, signs, hospitalization, structural heart disease evidence, echocardiographic data (LVEF criteria varies among studies), and NP levels for HFmrEF/HFpEF diagnosis.

**Table 3:** Specific diagnostic algorithm/criteria

Guideline	Diagnostic algorithm/criteria	
	Name	Description
ESC 2021 [1]	The simplified diagnostic approach	The simplified diagnostic approach starts with assessment of pre-test probability (clinical characteristics). The diagnosis should include the following: 1) Symptoms and signs of HF 2) A LVEF ≥ 50%^a^ 3) Objective evidence of cardiac structural and/or functional abnormalities consistent with the presence of LV diastolic dysfunction/ raised LV filling pressures, including raised NPs ^*a*^*Of note, patients with a history of overtly reduced LVEF (*≤ *40%), who later present with LVEF* ≥ *50%, should be considered to have recovered HFrEF or ‘HF with improved LVEF’ (rather than HFpEF)*
	H_2_FPEF	*Described in detail in the AHA/ACC/HFSA 2022 guideline*
	HFA-PEFF	*Described in detail in the HFA/ESC 2020 guideline*
HFA/ESC 2020 [26]	HFA-PEFF	A stepwise diagnostic process, the ‘HFA–PEFF diagnostic algorithm’ • Step 1 (P = Pre-test assessment) is typically performed in the ambulatory setting and includes assessment for: - HF symptoms and signs, typical clinical demographics (obesity, hypertension, diabetes mellitus, elderly, AF), and - Diagnostic laboratory tests, electrocardiogram, and echocardiography - In the absence of overt non-cardiac causes of breathlessness, HFpEF can be suspected if there is a normal LVEF, no significant heart valve disease or cardiac ischaemia, and at least one typical risk factor - Elevated natriuretic peptides support, but normal levels do not exclude a diagnosis of HFpEF • Step 2: (E: Echocardiography and Natriuretic Peptide Score) requires comprehensive echocardiography and is typically performed by a cardiologist - Measures include mitral annular early diastolic velocity (e′), LV filling pressure estimated using E/e′, left atrial volume index, LV mass index, LV relative wall thickness, tricuspid regurgitation velocity, LV global longitudinal systolic strain, and serum natriuretic peptide levels - Major (2 points) and Minor (1 point) criteria were defined from these measures. A score ≥ 5 points imply definite HFpEF; ≤ 1 point makes HFpEF unlikely. An intermediate score (2–4 points) implies diagnostic uncertainty, • Step 3 (F1: Functional testing) is recommended with echocardiographic or invasive haemodynamic exercise stress tests • Step 4 (F2: Final aetiology) is recommended to establish a possible specific cause of HFpEF or alternative explanations
DGK 2021 [17]	The simplified diagnostic approach	• The simplified diagnostic approach same as reported by the ESC 2021 guideline
AHA/ACC/HFSA 2022 [2]	H_2_FPEF	The H_2_FPEF score, integrates these predictive variables: • Obesity, AF, age > 60 years, treatment with ≥ 2 antihypertensive medications, echocardiographic E/e′ ratio > 9, and echocardiographic PA systolic pressure > 35 mm Hg • A weighted score based on these 6 variables was used to create the composite score ranging from 0 to 9. The odds of HFpEF doubled for each 1-unit score increase (odds ratio, 1.98; 95% CI: 1.74–2.30; P < 0.0001), with a c-statistic of 0.841. Scores < 2 and ≥ 6 reflect low and high likelihood, respectively, for HFpEF. A score between 2 and 5 may require further evaluation of hemodynamic with exercise echocardiogram or cardiac catheterization to confirm or negate a diagnosis of HFpEF The use of this H_2_FPEF score may help to facilitate discrimination of HFpEF from noncardiac causes of dyspnoea and can assist in determination of the need for further diagnostic testing in the evaluation of patients with unexplained exertional dyspnoea
JCS/JHFS 2021 [3]	H_2_FPEF	*Described in detail in the AHA/ACC/HFSA 2022 guideline*

*HF* heart failure, *H*_*2*_*FPEF* heavy, 2 or more hypertensive drugs, atrial fibrillation, pulmonary hypertension, elder age > 60, elevated filling pressures, *HFA-PEFF* heart failure association-pre-test assessment, echocardiography and natriuretic peptide score, functional testing, final aetiology, *LVEF* left ventricular ejection fraction, *ESC* European Society of Cardiology, *ACC* American College of Cardiology, *AHA* American Heart Association, *HFSA* Heart Failure Society of America, *JCS* Japanese Circulation Society, *JHFS* Japanese Heart Failure Society, *HFpEF* heart failure with preserved ejection fraction, *AF* atrial fibrillation, *DKG* German Society of Cardiology, *LV* left ventricular, *HFA* Heart Failure Association, *HFrEF* heart failure with reduced ejection fraction, *NPs* natriuretic peptides

### Prevalence

**Fig. 3:**
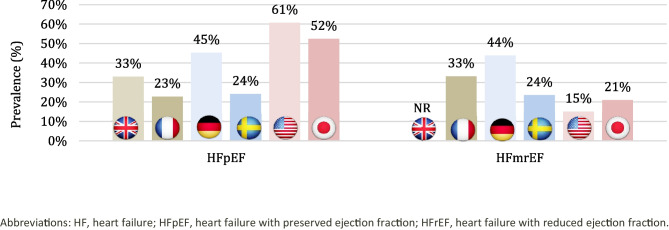
Most recent data on the prevalence of heart failure phenotypes in France, Germany, Sweden, the United Kingdom, Japan, and the United States [47, 59–64]

The prevalence of HFmrEF and/or HFpEF among HF patients was reported in 29 studies. The overall prevalence of HF has reportedly increased, with approximately 50% of symptomatic HF patients having HFmrEF/HFpEF [47, 59–63]. However, estimates of HFpEF prevalence varied among countries (Fig. 3) [47, 59–64]. A US-based single-center study involving 874 patients revealed the highest prevalence of HFpEF among patients with HF, reaching 61% [62]. In the same study, 15% of HF patients were diagnosed with HFmrEF. A similar trend was observed in the US-based National Cardiovascular Data Registry Practice Innovation and Clinical Excellence (NCDR PINNACLE) registry [65], where 56.5% of patients with HF had HFpEF and 7.5% had HFmrEF [65]. The regional distribution of HF phenotypes across the United States was heterogenous and may have reflected differences in the prevalence of main risk factors (including obesity, hypertension, or diabetes), with HFpEF and HFmrEF being most prevalent in the South [65]. Among the European countries of interest, the highest prevalence of HFpEF and HFmrEF in patients with HF was reported in Germany at 45% and 44%, respectively, as reported by Wenzel et al. [47]. Additionally, Gobel et al. reported a prevalence of 37% for HFpEF in the same country [66]. The lowest prevalence was reported in France (23% and 33%, respectively) and in Sweden (24% for both HFpEF and HFmrEF) [60, 61]. In Japan, HFpEF prevalence was high across patients hospitalized because of HF (43%) [63, 67]. In Japan additional prevalence data were reported in specific sub-populations showing 44.3% in patients on maintenance hemodialysis (44.3%) [63, 67]; 18.6% and 25% across HF adults with comorbid diabetes and HF [56]. Across studies with decompensated HF patients, HFpEF prevalence ranged between 43% [68] and 55.1% [69]. In Japan, similarly, as in other countries, the prevalence of HFmrEF was lower and ranged between 15 and 21% for HF patients overall [62, 70].

### Incidence

Recent data on the incidence of HFpEF and/or HFmrEF are scarce, with only 1 identified study conducted in the United States, using data limited to just 1 US district (a cohort of adult residents from Olmsted County, Minnesota). The cumulative incidence at 6 years was 11.7% for advanced HFpEF (defined as refractory HF symptoms despite attempts to optimize guideline-directed medical therapies) and 11.4% for HFmrEF [57].

### Mortality

Results for mortality among patients with HFpEF and/or HFmrEF were reported in 22 studies. The US-based studies showed varied mortality results due to differences in study design, patient characteristics, and outcome definitions. For HFpEF patients with similar baseline risks, the 1-year all-cause mortality ranged from 18.5% to 26.4% [71, 72]; patients with a higher baseline risk (admitted to intensive care) had a higher 1-year mortality risk (35.1% for HFpEF and 24.6% for HFmrEF) [73]. Patients with HFmrEF had a lower unadjusted risk of in-hospital death (8% vs 12%) than patients with HFpEF. However, after adjustment, the short-term mortality differences were not significant [73]. In Sweden, HFmrEF was associated with lower all-cause mortality compared with HFpEF (10.3 vs 13.2 events per 100 patient-years) [61]. However, in a study in France, there was no significant difference in mortality among HFmrEF and HFpEF patients hospitalized for congestive HF. In this study over a follow-up period of 2.17 ± 1.38 years, 41.3% of 383 deaths (158 deaths) were in patients with HFpEF, and 39.3% (108 deaths) were in patients with HFmrEF [74]. In a retrospective UK study covering 2 12-month periods, patients admitted with HF showed lower overall mortality in the 2015/2016 cohort compared to 2012/2013, especially in HFpEF cases (13.3% vs 16.3%, *P* = 0.435). There was no significant difference in in-hospital mortality between the 2 cohorts for HFpEF (*P* = 0.472). Notably, for HFpEF, 30-day post-discharge mortality decreased from 8.9% (2012/2013) to 3.1% (2015/2016) (*P* = 0.032) [75]. Improved mortality was prominent among cardiology ward patients, possibly due to optimised fluid status and extended inpatient stays [75]. The Swedish registry data (SwedeHF) [44] revealed that HFpEF patients on beta-blockers had lower mortality rates at 1, 3, and 5 years (16%, 37%, and 53%, respectively) with an incidence rate of 157 per 1000 patient-years (95% CI: 153–162) compared with non-beta-blocker users. The non-beta-blocker users had mortality rates of 22%, 47%, and 62% at 1, 3, and 5 years, respectively, with an incidence rate of 209 per 1000 patient-years (95% CI: 197–221) [44]. A Swedish study revealed that hypertension, atrial fibrillation, and ischemic heart disease were the main factors for mortality in HFpEF. Being of a younger age and having a low comorbidity burden were associated with lower mortality rates [36].

In Japan, for HFpEF, the in-hospital mortality rate was 8%; for HFmrEF, it was 6% [63]. After discharge, the mortality rate ranged from 16% to 24.7% [63, 76].

### Comorbidities

The most common comorbidities among patients with HFmrEF/HFpEF are hypertension, atrial fibrillation, coronary artery disease (CAD), diabetes, obesity, chronic kidney disease (CKD), and chronic obstructive pulmonary disease (COPD). In a large US registry (PINNACLE; 2008–2016) with 697,542 patients with HF, including 324,387 patients with HFpEF and 56,527 with HFmrEF [65], the most prevalent comorbidities among HFpEF patients were hypertension (79.1%), CAD (55.5%), atrial fibrillation (AF) or atrial flutter (AFL) (34.4%), and diabetes (25.7%). Patients with HFmrEF had a higher prevalence of CAD, peripheral artery disease, AF or AFL, CKD, diabetes, and prior myocardial infarction compared with those who had HFrEF or HFpEF (*P* < 0.001) [65]. In a 2013 to 2016 US-based outpatient registry of patients with diabetes and prediabetes who were prescribed ≥ 1 glucose-lowering medication and diagnosed with HF (55.5% with LVEF ≥ 50%), CV comorbidities were common: 87.3% had hypertension, 59.0% had CAD, and 37% had AF/AFL [77]. In a large nationwide Swedish registry (2000–2018), collecting data mainly from secondary care inpatients, outpatient wards and clinics, and primary care settings for clinician-judged HF, the most frequently reported comorbidities among patients with HF at follow-up in specialty care versus primary care were as follows: hypertension (62.2% vs 76.6%), AF (54.8% vs 63.3%), ischemic heart disease (54.3% vs 56.7%), kidney disease (44.3% vs 61.7%), and anemia (33% vs 40.8%) [61]. The prevalence of major comorbidities was slightly higher in patients with HFpEF compared to HFmrEF, in both specialty care and primary care [61]. In the largest Japanese prospective observational study (2010–2018), the most commonly reported comorbidities in patients with HFpEF and HFmrEF were hypertension and dyslipidemia, followed by CKD, anemia, and sleep-disordered breathing [41].

### Clinical burden

#### Efficacy in randomized controlled trials

The efficacy of interventions in patients with HFmrEF/HFpEF has been largely studied in RCTs. The most commonly reported outcome in RCTs was a composite of CV death or HF hospitalization, but definitions varied, affecting interpretation. The main differences were related to the inclusion of first/recurrent hospitalizations and HF with or without urgent visits. Additionally, some studies categorized deaths from undetermined causes as CV deaths (eg, EMPEROR-Preserved) [78], although others excluded them (eg, DELIVER) [79]. Overall, interventions reduced the HF hospitalization risk, but the effect on mortality was not significant (Table 4). In large, long-term, HF-specific RCTs, SGLT-2is—mainly dapagliflozin (DELIVER) [79] and empagliflozin (EMPEROR-Preserved) [78]—significantly reduced the risk of a composite of CV death or hospitalization for HF compared with controls [79, 80]. This effect was mainly driven by the risk reduction for HF hospitalization. Overall, SGLT-2is [dapagliflozin [79] and empagliflozin [81, 82] and spironolactone therapy significantly reduced HF hospitalization compared with controls; sacubitril/valsartan had no effect on HF events [35]. Candesartan moderately reduced HF hospitalizations [unadjusted hazard ratio (HR): 0.85 (95% CI: 0.72–1.01), *P* = 0.072; adjusted HR: 0.84 (95% CI: 0.70–1.000), *P* = 0.047] in the HFpEF population, with fewer HF hospitalizations compared with the placebo group (230 vs 279, *P* = 0.017) [83]. In the perindopril in elderly people with chronic heart failure (PEP-CHF) study, perindopril lowered the HF hospitalization risk in the first year [HR: 0.63 (95% CI: 0.41–0.97), *P* = 0.033], but it did not have this effect throughout the follow-up period (mean: 26.2 months) [84]. No treatment significantly reduced the CV death risk, regardless of the definition used. Most RCTs analyzed subgroups based on demographics, medical history, or prior treatments for the primary outcome only, which differed among studies. The overall effect was generally consistent across subgroups. In the EMPEROR-Preserved study, conducted in patients with EF > 40%, the empagliflozin group had a significantly lower risk of CV death or worsening HF events (hospitalization for HF or an urgent HF visit requiring intravenous therapy) compared with the placebo group. The benefit increased when only patients with LVEF < 60% were considered [82]. In PARAGON-HF, the sacubitril/valsartan group showed a significantly lower risk of CV death or HF hospitalization compared with patients administered valsartan alone, with greater benefit afforded to patients with an LVEF of 45% to 57% and women [85]. In TOPCAT, spironolactone's effect varied based on the randomization stratum and prior HF hospitalization. This effect was better among patients not hospitalized for HF in the year prior to study enrollment. Post hoc analysis showed significant regional differences in event rates for the primary outcome (CV death, aborted cardiac arrest, or hospitalization for HF), with a significant risk reduction in the Americas (in the United States, Canada, Brazil, and Argentina) but not in Russia or Georgia [86].

**Table 4:** Main results from included randomized controlled trials

Study	Population	Follow-up	Interventions	Sample size	Impact of intervention on outcome measure [HR (95% CI), P-value]			
					CV death or HHF	CV death	HHF	Composite renal outcome
SGLT-2is								
DECLARE-TIMI [53] (NCT01730534)	Adult patients with HF, T2D, and LVEF ≥ 45%	NR	Dapagliflozin vs placebo	4796	Neutral 0.88 (0.66; 1.17)	Neutral 1.41 (0.93; 2.13)	Neutral 0.72 (0.5; 1.04)	**Positive** 0.52 (0.3; 0.9)
DELIVER [79] (NCT03619213)	Adult patients with HF (NYHA II-IV) and LVEF > 40%	Median FU: 2.3 years	Dapagliflozin vs placebo	6263	**Positive** 0.82 (0.73; 0.92), p < 0.01	Neutral 0.88 (0.74; 1.05)	**Positive** 0.77 (0.67; 0.89)	NR
EMPEROR-Preserved [82] (NCT03057951)	Adults with CHF (NYHA II-IV) and LVEF > 40%	Median FU: 26.2 months	Empagliflozin vs placebo	5988	**Positive** 0.79 (0.69; 0.9), P = 0.0003	Neutral 0.91 (0.76; 1.09), P = 0.295	**Positive** 0.71 (0.6; 0.83), P < 0.0001^a^	Neutral 0.95 (0.73; 1.24)^b^
SCORED [34] (NCT03315143)	Subpopulation: adult patients with T2D, CKD, risk of CV disease, and LVEF ≥ 50%	Up to 30 months	Sotagliflozin vs placebo	1667	**Positive** 0.72 (0.52; 0.99)	NR	NR	NR
CANONICAL [132] (jRCTs051180030)	Adult patients with HF (NYHA II-III) and LVEF ≥ 50% with T2D	24 weeks	Canagliflozin vs standard diabetic therapy	82	NR	NR	Neutral P = 1.00	NR
VERTIS-CV [51] (NCT01986881)	Adult patients with T2D and HF; LVEF > 45%	Mean FU: 3.5 years	Ertugliflozin vs placebo	1007	Neutral 0.92 (0.61; 1.39)	Neutral 1.08 (0.64; 1.80)	Neutral 0.7 (0.39; 1.26)	NR
ARBs								
CHARM-Preserved [83] (NCT00634712)	Adult patients with CHF (NYHA II-IV) and LVEF > 40%	Median FU: 36.6 months	Candesartan vs placebo	3023	Neutral 0.89 (0.77; 1.03), p = 0.118	Neutral 0.99 (0.9; 1.22), P = 0.918	Neutral 0.85 (0.72; 1.01), P = 0.072	NR
I-PRESERVE [134] (NCT00095238)	Adult patients with HF (NYHA II-IV) and LVEF ≥ 45%	Mean FU: 49.5 months	Irbesartan vs placebo	4128	Neutral 0.96 (0.84; 1.09), p = 0.51^c^	Neutral 1.01 (0.86; 1.18), P = 0.92	Neutral 0.95 (0.85; 1.08), P = 0.44^d^	NR
ARNIs								
PARAGON-HF [85] (NCT01920711)	Patients with chronic HF (NYHA II-IV) and LVEF ≥ 45%	Median FU: 35 months	Sacubitril/valsartan vs valsartan	4796	Neutral RR = 0.87 (0.75; 1.01), P = 0.06	Neutral 0.95 (0.79; 1.16)	Neutral RR = 0.85 (0.72; 1), P = 0.072	**Positive** 0.5 (0.33; 0.77)
Feng 2022[35] (ChiCTR2000031485)	Adult patients with HFpEF (NYHA II-III) and LVEF ≥ 40%	After 10 weeks of treatment	Sacubitril/valsartan vs basic treatment	78	**Positive** P = 0.013^e^	NR	NR	NR
MRAs								
TOPCAT [86] (NCT00094302)	Adult patients with symptomatic HF and LVEF ≥ 45%	Mean FU: 3.3 years	Spironolactone vs placebo	3445	Neutral 0.89 (0.77; 1.04), P = 0.14^f^	Neutral 0.9 (0.73; 1.12), P = 0.35	**Positive** 0.83 (0.69; 0.99), P = 0.04	NR
ACE-Is								
PEP-CHF [84]	Elderly patients (> 70 years old) with HF and LVEF ≥ 50%, treated with diuretics	Mean FU: 26.2 months	Perindopril vs placebo	850	Neutral 0.92 (0.7; 1.21), p = 0.545^ g^	Neutral 0.86 (0.61; 1.2), P = 0.375	**Positive** / No effect^h^ 0.63 (0.41; 0.97), P = 0.033 0.86 (0.61; 1.2), P = 0.375	NR

*ACE-I* angiotensin-converting enzyme inhibitor, *ARB* angiotensin receptor blocker, *ARNIs* angiotensin receptor-neprilysin inhibitor, *CHF* congestive heart failure, *CV* cardiovascular, *FU* follow-up, *HF* heart failure, *HHF* hospitalization for heart failure, *HR* hazard ratio, *LVEF* left ventricular ejection fraction, *MRA* mineralocorticoid antagonist, *NR* not reported, *NYHA* New York Heart Association, *RR* rate ratio, *SGLT-2i* sodium-glucose co-transporter 2 inhibitor, *T2D* type 2 diabetes

^a^Time to first hospitalization

^b^Composite renal outcome (time to first occurrence of CD, renal transplantation, sustained decrease of ≥ 40% in eGFR, and sustained eGFR of < 10 or < 15 for patients with < 30 or ≥ 30 mL/min/1.73 m2 at baseline, respectively)

^c^HF death or HHF

^d^Hospitalization for a protocol-specified CV cause including HF

^e^Composite of CV death or CV events (worsening HF, MI, or CV rehospitalization)

^f^CV death or HHF or aborted cardiac arrest

^g^Composite of all-cause mortality or unplanned HHF (worsening HF)

^h^At 1 year/ at mean follow-up of 26.2 months

#### Real-world effectiveness

There is a limited availability of RWE regarding patients with HFpEF and/or HFmrEF. The existing data mainly came from subgroup analysis, so caution is advised in their interpretation. There was significant heterogeneity among the included studies, particularly in the definitions of HFmrEF/HFpEF, baseline characteristics, comorbidity burden, and outcome definition and follow-up. Most studies focused on the clinical burden in patients treated with standard HF treatments, without focusing on any specific drugs.

The real-world data indicated high morbidity with increased rates of hospitalization. According to Afzal et al., in the United States, hospitalizations for HF increased from 45,148 in 2004 to 297,125 in 2016. The number of diastolic HF cases (HFpEF) increased between 2007 and 2008 but decreased significantly in 2017 because of changes in HF coding [87]. Additionally, Clark et al., found a significant increase in hospitalizations due to HFpEF from 189,260 in 2008 to 495,095 in 2018 [88]. Over time, hospitalizations due to HFpEF involved younger patients (from a mean age of 78 years in 2008 to 77 years in 2018) and were less common among female patients (from 65.3% to 60.3% in 2018; *P* < 0.001 for all). The prevalence of comorbidities also rose among HFpEF hospitalizations, including diabetes (43.0% in 2008 to 51.6% in 2018), obesity (14.2% to 32.8%), and obstructive sleep apnea (9.2% to 19.5%) (*P* < 0.001 for all). Reinhardt et al. studied hospitalization trends in HF and AF patients using the National Inpatient Sample (NIS) between 2008 and 2017. Among HFpEF patients, there were 3,117,059 admissions, with the percentage of HFpEF hospitalizations with comorbid AF rising from 38.0% in 2008 to 49.1% in 2017 [89]. Patients with HFpEF and AF with comorbid hypertension had the highest hospitalization rates. Results from a US cohort study (2010–2019) showed increasing hospitalizations for worsening HF from 0.6 to 1.0 per 100 hospitalizations per year for HFmrEF. For HFpEF, hospitalizations increased from 2.6 to 3.9 per 100 hospitalizations per year [90]. In patients from the SwedeHF registry who had HFpEF, beta-blockers did not impact HF hospitalizations at 5 years [42% with beta-blockers vs 43% without; unadjusted HR: 0.97 (95% CI: 0.90–1.05)]. In the matched cohort, no significant association was found between beta-blockers and HF admissions [HR: 0.95 (95% CI: 0.87–1.05)] [44]. However, beta-blockers were associated with a lower risk of all-cause hospital admissions at 1 year and 5 years (56% and 88% with beta-blockers; 60% and 91% without) [44].

Real-world data for CV deaths in patients with HFpEF and/or HFmrEF have not been widely reported, making comparisons with RCTs difficult because of study design and baseline differences. A cluster analysis identified clinically distinct HFpEF clusters, with the highest CV death incidence and hazard ratio in the cluster including older patients with multiple CV comorbidities and hypertension [36]. A US study comparing classification approaches for elderly patients with HF found similar 1-year CV death rates in HFpEF cases, with slightly different contributions to overall mortality based on the approach used [91]. A Swedish study found that beta-blockers significantly reduced the risk of CV mortality [HR: 0.8 (95% CI: 0.73–0.87), P < 0.001] in patients with HFpEF. At 5 years, CV death was reported in 40% (95% CI: 37–43) of non-beta-blocker users and 35% (95% CI: 33–36) of beta-blocker users (48). In Japan, a study showed lower CV deaths in tolvaptan responders with HFpEF (5.9%) compared with non-responders (18%); the difference was not significant compared with patients with HFrEF (*P* = 0.288 for responders; *P* = 0.245 for non-responders) [76].

#### Safety in randomized controlled trials

Overall, 21 studies reported safety outcomes in populations of patients with HFpEF and/or HFmrEF. The investigated treatments for HFpEF (with or without HFmrEF), including SGLT-2is, MRAs (spironolactone), ARNIs (sacubitril/valsartan), ACE-Is, and ARBs, exhibited a generally good safety profile. Specific side effects included genital and urinary tract infections, hypotension, and fractures for SGLT-2is or hyperkalemia, worsening of renal function, and anemia for spironolactone. The percentage of patients discontinuing treatment was comparable between study arms in all studies except one, in which a numerically higher percentage of patients discontinued ramipril treatment compared with diuretics (13.3% vs 6%) [80]. The overall rates of adverse events (AEs) were similar among the treated patients and the comparator groups, except for 1 trial of sacubitril/valsartan, which reported a significantly higher percentage of total AEs in the sacubitril/valsartan group compared with the background medication-based comparator group [32]. The most frequent AEs during sacubitril/valsartan treatment were hypotension and hyperkalemia.

#### Real-world safety

Real-world safety data were limited (reported in 3 studies) but indicated higher rates of hyperkalemia hospitalizations among patients with HFpEF who started spironolactone between 2013 and 2018 (crude incidence rate: 9.8 per 1000 patient-years) [92].

### Humanistic burden

The impact of HFmrEF/HFpEF on patients' HRQoL is substantial; it is associated with a wide range of symptoms and functional limitations that affect daily activities, physical abilities, and emotional well-being. The Kansas City Cardiomyopathy Questionnaire (KCCQ) was the most commonly used tool across the identified studies, for both RCTs (8 studies) and RWE (7 studies). The KCCQ has been qualified by the US Food and Drug Administration (FDA) as a clinical outcome assessment for HF and is recommended for measuring care quality. Regulatory bodies, including the European Medicines Agency and FDA, have utilized it in drug assessments [93, 94]. The KCCQ scale is considered a reliable and sensitive to clinical change tool, that has been validated for both HFrEF and HFpEF patients, with a 5-point improvement considered a minimal clinically important difference [95, 96]. Therefore, we focused our results on the KCCQ tool. The KCCQ is a 23-item, self-administered questionnaire that quantifies seven domains impacting HF patients' lives, including their physical and social limitations, symptom frequency and severity, quality of life, recent changes in symptom status, and self-efficacy. The symptom frequency and symptom burden are merged into a total symptom score, which combined with the physical limitation domain create an overall clinical score. An overall summary score comprising the total symptom score, physical limitation, quality of life and social limitation domains. Scores for each domain range from 0 to 100, with higher scores indicating a lower symptom burden and better quality of life. The scores are categorized to represent health status as follow: 0 to 24 (very poor to poor), 25 to 49 (poor to fair), 50 to 74 (fair to good), and 75 to 100 (good to excellent) [97].

#### Health-related quality of life in randomized controlled trials

Overall, 15 trials reported results for HRQoL in the population of patients with HFpEF and or HFmrEF. The change in the KCCQ total symptom score from baseline to month 8 showed that dapagliflozin provided benefits compared with the placebo for symptoms of HF [win ratio^1^: 1.11 (95% CI: 1.03–1.21)], *P* = 0.009) [79]. Another study revealed that dapagliflozin significantly improved the KCCQ clinical summary score (*P* = 0.001), the total symptom score (*P* = 0.003), and the physical limitations score (*P* = 0.026) compared with placebo; patients treated with dapagliflozin rather than placebo had a ≥ 5-point improvement in overall KCCQ score [adjusted odds ratio (OR): 1.73 (95% CI: 1.05–2.85), *P* = 0.03] [80]. Similarly, empagliflozin improved HRQoL, including KCCQ clinical summary, total symptom, and overall summary scores compared with the placebo at 12 weeks; this effect was durable up to 52 weeks. This finding was supported by a responder analysis. At 12 weeks, patients on empagliflozin had higher odds of improvement ≥ 5 points [OR: 1.23 (95% CI, 1.10–1.37)], ≥ 10 points [OR: 1.15 (95% CI, 1.03–1.27)], and ≥ 15 points [OR: 1.13 (95% CI, 1.02–1.26)], as well as lower odds of deterioration ≥ 5 points in KCCQ clinical summary score [OR: 0.85 (95% CI, 0.75–0.97)] compared with placebo. A similar pattern was seen at 32 and 52 weeks, and the results were consistent for the total symptom and overall summary scores [78]. In the PARALAX trial, an improvement in KCCQ score was observed; however, no significant differences between sacubitril/valsartan and the control group were reported in the mean change in the KCCQ clinical summary score from the baseline. The proportion of patients experiencing an improvement or decrease by ≥ 5 points was similar in both treatment groups [32]. In PARAGON, a decrease in the KCCQ clinical summary score was observed and the mean change at 8 months was 1.0 point higher in the sacubitril/valsartan group compared with the valsartan group [85]. A higher percentage of patients in the sacubitril/valsartan group than in the valsartan group had an improvement of ≥ 5 points in their KCCQ clinical summary score (33.0% vs 29.6%; OR: 1.30; 95% CI, 1.04–1.61) [85]. The results showed that spironolactone led to better patient-reported outcomes. In the TOPCAT trial, the spironolactone group had a significantly higher mean change in KCCQ compared with the placebo group at months 4 (*P* = 0.002) and 12 (*P* = 0.02), but this effect was not seen at the end of the study. At 4 months, spironolactone also improved the KCCQ clinical summary and symptom scores compared with placebo, but these improvements did not persist beyond 4 months. There were no significant differences among the treatment groups in the other KCCQ domains (social interference, physical scores, and quality of life) during the follow-up period [98].

#### Health-related quality of life in real-world evidence studies

Overall, 11 RWE studies reported HRQoL results in patients with HFmrEF/HFpEF. The real-world data indicated a decline in overall KCCQ scores in patients with HFpEF compared with HFmrEF. In the BIOSTAT-CHF study, patients with HFpEF reported more physical limitations, increased symptom frequency, and greater symptom burden, as well as having more social limitations [99]. Additionally, it was observed that most non-cardiac comorbidities (diabetes mellitus, obesity, thyroid dysfunction, CKD, stroke, COPD, peripheral artery disease, and anemia) were associated with a significant decline in the overall KCCQ score. For patients with HFmrEF or HFpEF, each comorbidity—except for peripheral artery disease in HFpEF—was associated with a decline in the score. For HFmrEF, all comorbidities except for CKD (mean difference of 4.48 points; 4.96 points for stroke) had minimal clinically important differences; for HFpEF, the only comorbidities with a minimal clinically important difference were COPD (mean difference of 10.8 points) and thyroid dysfunction (mean difference of 4.9 points) [99]. One study revealed a correlation between overall KCCQ summary scores and NYHA class. Higher scores were observed for lower NYHA classes and vice versa in HFpEF (r =  − 0.62, *P* < 0.001) patients. Similarly, KCCQ total symptom domain scores showed a significant correlation with NYHA class in HFpEF (r =  − 0.61, *P* < 0.001) patients [96].

### Economic burden

Overall, 4 RWE publications reported outcomes for direct costs in patients with HFpEF and 10 reported outcomes for direct resource use for hospitalizations due to HFpEF and/or HFmrEF. Nevertheless, the available data are primarily limited to the United States. A study conducted in the United States from 2012 to 2018 revealed that the average per-patient monthly cost for healthcare was $7482. This cost was primarily driven by high rates of inpatient and outpatient visits, with costs of $4668 for inpatient stays, $2318 for outpatient visits, and $495 for medications [100]. Another US study conducted from 2008 to 2018 indicated that although the number of hospitalizations increased, the median inpatient costs of hospitalization due to HFpEF decreased from $9071 in 2008 to $8306 in 2018. This increase in the number of hospitalizations was related to changes in HF coding practices over time; however, the decrease in the inpatient costs of HF hospitalization may be due to improved management of HF exacerbations, enhanced outpatient management, and new therapeutic agents [88]. A comparison of healthcare resource use among HFmrEF and HFpEF patients in the United States (2007–2018) showed that the length of stay in the cardiac intensive care unit (CICU) and in hospital was longer in patients with HFmrEF than HFpEF (median length: 8.5 vs 6.9 days and 2.8 vs 2 days, respectively) [73]. In the United States, the length of stay was relatively stable over time; the length of hospital stays per patient ranged between 4 and 5 days per year for adult patients with HFpEF [100]. In Japan, the length of stay was longer than in the United States and ranged between 17 and 38 days [63, 101].

### Pharmacologic treatment

#### Guideline-directed pharmacologic therapy 

Guideline-directed pharmacologic therapy for HFmrEF and HFpEF focuses on reducing congestion symptoms with diuretics and treating underlying comorbidities (Table 5). Limited evidence exists of specific treatments for HFmrEF, and no prospective RCTs have been conducted exclusively for HFmrEF patients. Commonly considered drugs for HFmrEF treatment include diuretics, ACE-Is, ARBs, beta-blockers, MRA, and ARNIs (Table 5). Recent treatment options for HFmrEF/HFpEF include sacubitril/valsartan (an ARNI), empagliflozin, and dapagliflozin (SGLT-2is). In 2021, the FDA approved sacubitril/valsartan's indication extension, based on the PARAGON‐HF trial, to include certain HFpEF patients with reduced ejection fractions. Now, its use is indicated for patients with chronic HF, and the benefits are most clearly seen in patients with a below normal LVEF, although the exact definition of a normal LVEF has not been provided [10, 102, 103]. In Europe, sacubitril/valsartan is currently approved only for patients with HFrEF [104]. Empagliflozin and dapagliflozin were initially approved for type 2 diabetes [6–9]; later, they were also approved for HFrEF [104, 105]. Subsequently, in 2022, empagliflozin’s indication was extended in Europe and the United States, making it the first therapy approved for adults with HFmrEF/HFpEF [106]. This was followed by dapagliflozin’s approval in the United Kingdom (2022) [107], Europe (2023) [8], and the United States (2023) for symptomatic chronic HF, including HFmrEF/HFpEF, in adult patients [9, 108]. In Japan, sacubitril/valsartan, dapagliflozin, and empagliflozin were also approved for use in the broad chronic HF population, but whether there are any limitations on HFrEF/HFmrEF/HFpEF patients in their indications has not been clearly stated [109, 110]. Clinical guidelines are beginning to incorporate SGLT-2is and ARNI recommendations for HFmrEF/HFpEF based on new evidence. American Heart Association (AHA), American College of Cardiology (ACC), and Heart Failure Society of America (HFSA) 2022 guidelines in the United States recommended SGLT-2is based on the EMPEROR-Preserved study for HFmrEF/HFpEF [2]. Multiple other guidelines, with similar classes of recommendation and levels of evidence, recommended an ARNI (sacubitril/valsartan) based on PARAGON-HF and combined PARADIGM-HF/PARAGON-HF analyses for HFmrEF/HFpEF (Table 6).

**Table 5:** Overview of drug classes recommended by clinical guidelines for heart failure with preserved ejection fraction/heart failure with mildly reduced ejection fraction by treatment guidelines

Guideline		Drug classes recommended/considered by guidelines	
		HFmrEF	HFpEF
Europe	HFA/ESC 2020 [26]	NR	NR
	ESC 2021 [1]	Diuretics, ACE-I, ARBs, BB, MRA, ARNI	Diuretics, drugs used to treat comorbidities
	ESH 2021 [30]	NR	Diuretics, MRAs, ARNi
France	HAS 2014 [18]	NR	Diuretics, drugs used to treat comorbidities
	HAS 2015 [29]	NR	
	SFGG 2021 [28]	NR	
Germany	NDMG 2018 [27]	Diuretics, ACE-I, ARBs, BB, MRAs	Diuretics, drugs used to treat comorbidities
	NVL 2019 [23]	Diuretics, ACE-I, ARBs, BB, MRAs, ARNI	
	DGK 2021 [17]	Diuretics, ACE-I, ARBs, BB, MRAs, ARNI, SGLT-2i, Ivabradine	
	IQWiG 2021 [19]	NR	NR
Sweden	SMA 2015 [25]	NR	Diuretics, drugs used to treat comorbidities
	NBHW 2018 [21]	NR	
	SKS 2021 [24]	Diuretics, ACE-I, ARBs, BB, MRAs, IV iron	
	LOK 2022 [20]	Diuretics, ACE-I, ARBs, BB, MRAs, ARNI, SGLT-2i	
United Kingdom	NICE 2018 [22]	NR	Diuretics
	CaReMeUK-HF 2022 [31]	NR	Diuretics
United States	AHA/ACC/HFSA 2022 [2]	Diuretics, SGLT2i, ARNi, ACE-I, ARBs, MRA	Diuretics, SGLT-2i, PDE5i, ACE-I, ARBs, MRA, ARNI
Japan	JCS/JHFS 2021 [3]	Diuretics, ARNi, ACE-I	Diuretics, drugs used to treat comorbidities

*AHA* American Heart Association, *ARB* angiotensin receptor blocker, *ARNI* angiotensin receptor-neprilysin inhibitor, *BB* beta-blockers, *CaReMeUK-HF* British Cardiovascular Society, *DKG* German Society of Cardiology, *ESH* European Society of Hypertension, *ESC* European Society of Cardiology, *HAS* French National Authority for Health, *HFmrEF* heart failure with mildly reduced ejection fraction, *HFpEF* heart failure with preserved ejection fraction, *HFSA* Heart Failure Society of America, *IQWiG* Independent Institute for Quality and Efficiency in Health Care, *JCS* Japanese Circulation Society, *JHFS* Japanese Heart Failure Society, *LOK* Pharmaceutical committees' national network, *MRAs* mineralocorticoid antagonists, *NBHW* National Board of Health and Welfare, *NDMG* National Disease Management Guideline, *NICE* National Institute for Health and Care Excellence, *NVL* National Care Guideline, *PDE5i* phosphodiesterase type 5 inhibitor, *SGLT-2i* sodium-glucose co-transporter 2 inhibitor, *SFGG* French Society of Geriatrics and Gerontology, *SMA*, Swedish Medicines Agency, *NR* not reported

**Table 6:** Overview of specific clinical guideline recommendations for sodium-glucose cotransporter-2 inhibitors and angiotensin receptor/neprilysin inhibitors for heart failure with preserved ejection fraction/heart failure with mildly reduced ejection fraction treatment

Guideline		Population	Drug class	COR	LOE	Recommendations	Referenced studies that support the recommendations
US	**AHA/ACC/HFSA 2022 **[2]	HFmrEF/HFpEF	SGLT-2i	2a (moderate)^a^	B-R^e^	In patients with HFmrEF/HFpEF, SGLT-2is can be beneficial in decreasing HFH and CV mortality	EMPEROR-Preserved (NCT03057951)
		HFpEF	ARNI	2b (weak)^b^	B-R^e^	In selected patients with HFpEF, ARNIs may be considered to decrease hospitalizations, particularly among patients with LVEF on the lower end of this spectrum	PARAGON-HF (NCT01920711)
		HFmrEF	ARNI	2b (weak)^b^	B-NR^f^	Among patients with current or previous symptomatic HFmrEF (LVEF, 41%-49%), ARNIs may be considered to decrease hospitalizations, particularly among patients with LVEF on the lower end of this spectrum	Combined analysis from two trials: PARADIGM-HF (NCT01035255) and PARAGON-HF (NCT01920711)
Europe	**ESC 2021 **[1]	HFpEF	ARNI	NR	NR	NR	NR
		HFmrEF	ARNI	IIb^c^	C^g^	Sacubitril/valsartan may be considered for patients with HFmrEF to reduce the risk of HF hospitalization and death	Combined analysis from two trials: PARADIGM-HF (NCT01035255) and PARAGON-HF (NCT01920711)
	**ESH 2021 **[30]	HFpEF	SGLT-2i	NR	NR	The results of upcoming RCTs shall provide more evidence on the role of these drugs in patients with HFpEF with or without diabetes	NR
		HFpEF	ARNI	NR	NR	ARNIs should be considered as a replacement for conventional RAS blockers in HFpEF patient groups (such as women and those with an LVEF at the lower end of the HFpEF spectrum) to reduce HF hospitalizations	PARAGON-HF (NCT01920711)
		HFmrEF	ARNI	NR	NR	NR	NR
Japan	**JCS/JHFS 2021 **[3]	HFpEF	ARNI	IIb^c^	B^h^	Administration of ARNI for HFpEF may be considered	PARAGON-HF (NCT01920711)
		HFmrEF	ARNI	IIa^d^	B^h^	A switch from ACE inhibitors (or ARBs) to ARNIs should be considered for NYHA class II or greater in HFmrEF patients treated with diuretics	Combined analysis from two trials: PARADIGM-HF (NCT01035255) and PARAGON-HF (NCT01920711)
Sweden	**LOK 2022 **[20]	*Regulatory authorities have not yet approved SGLT-2i for HFpEF*					

*ACC* American College of Cardiology, *AHA* American Heart Association, *ARNI* angiotensin receptor-neprilysin inhibitor, *COR* class of recommendation, *ESC* European Society of Cardiology, *HFH* heart failure hospitalizations, *HFmrEF* heart failure with mildly reduced ejection fraction, *HFpEF* heart failure with preserved ejection fraction, *JCS* Japanese Circulation Society, *JHFS* Japanese Heart Failure Society, *LOE* level of evidence, *NR* not reported, *SGLT-2i* sodium-glucose co-transporter 2 inhibitor

^a^Class 2a (moderate): benefit > risk, suggested phrases for writing recommendations; is reasonable, can be useful/effective/beneficial

^b^Class 2b (weak): benefit ≥ risk, suggested phrases for writing recommendations; may/might be reasonable, may/might be considered, usefulness/effectiveness is unknown/unclear/uncertain or not well-established

^c^Class IIb: usefulness/efficacy is less well established by evidence/opinion; wording to use, may be considered

^d^Class IIa: there is high probability of efficacy/usefulness based on evidence and opinion

^e^Level B-R (randomized): moderate-quality evidence from 1 or more RCTs, meta-analysis of moderate-quality RCTs

^f^Level B-NR (non-randomized): moderate-quality evidence from 1 or more well-designed, well-executed non-randomized studies, observations studies, or registry studies, meta-analysis of such studies

^g^Level of evidence C: consensus of opinion of the experts and/or small studies, retrospective studies, or registries

^h^Level of evidence B: demonstrated by a single randomized clinical trial or large nonrandomized studies

#### Real-world practice 

Studies on HFpEF and/or HFmrEF patients reported real-world treatment patterns as percentages for different therapies [13, 36–40, 43, 44, 46, 47, 49, 50, 60, 62, 63, 65, 68, 69, 76, 77, 90–92, 100, 111]. Commonly used medications included beta-blockers, renin-angiotensin system inhibitors (RASIs), diuretics (loop or thiazide), ACE-Is/ARBs (in combination or separately), anti-coagulants, and calcium channel blockers, as well as statins and MRAs. SGLT-2i use in real-life has rarely been reported. No significant differences in treatment patterns were found among patients with HFpEF and HFmrEF [13, 36–40, 43, 44, 46, 47, 49, 50, 60, 62, 63, 65, 68, 69, 76, 77, 90–92, 100, 111].

## Discussion

This TLR provides up-to-date data on the epidemiology, burden of illness, and current pharmacologic landscape of HFmrEF/HFpEF, alongside identifying unmet needs and knowledge gaps.

The literature indicates a lack of consensus regarding the characterization and diagnosis of HFpEF and HFmrEF, with variation in diagnostic criteria observed across scientific society guidelines and clinical trials. This variation partly arises from an incomplete understanding of disease pathophysiology and the heterogenous nature of the disease which involves a multitude of contributing risk factors, causes, and phenotypic manifestations [26, 112]. Our results reveal a concerning gap in understanding regarding the predictors and risk factors of HFmrEF/HFpEF, which confirms the needs for additional research to better understand such factors and natural history. Given the persisting challenges in HFpEF diagnosis, various scientific societies have proposed specific diagnostic criteria, tools, and algorithms, which are referenced in key guidelines (although not discussed within the context of HFmrEF). However, the additional validation of these scoring tools and their practical applicability in routine clinical practice are still subject to discussion [1–3, 17, 26]. To date, RCTs mainly refer to a documented diagnosis of symptomatic HF with typical symptoms/signs of HF combined with LVEF thresholds of ≥ 40%, ≥ 45% or ≥ 50%, evidence of structural heart disease or hospitalization for HF within 12 months, as well as elevated NT-proBNP threshold. The potential impact of evolving diagnostic criteria on the definition of RCT populations in the future remains to be evaluated. In addition, inconsistencies exist in the definition of subgroups of patients whose EF transitioned among guidelines. These variations may contribute to the complexity of patient classification and tailored management strategies. On the other hand, real-world results indicate an increase in HF hospitalizations, especially in cases of HFpEF, possibly because of changes in coding practices, emphasizing the need for an accurate HF classification [87].

Approximately 50% of patients with symptomatic HF are reported to have HFpEF, while HFmrEF is less common. The recent incidence and epidemiology trends data identified for the selected geographical scope were relatively scarce, because most epidemiology data were reported before 2016, cut-off date of our review. Moreover, most epidemiology data refer to HFpEF rather than HFmrEF.

Epidemiology data were found to be heterogeneous, reflecting the heterogeneity of HFmrEF/HFpEF disease. Furthermore, estimates of HFpEF and HFmrEF prevalence varied among countries, and caution is advised when interpreting these estimates because of the variations in definitions and study characteristics across different regions and populations. Another important aspect that may have influenced the prevalence estimates is the changes in the definition of HF provided by the guidelines over time [47]. A study in Germany assessed the impact of these changes between the 2016 ESC HF guidelines and the 2021 ESC guidelines, finding notable differences in prevalence estimates for HFpEF and HFmrEF. This review found that HF prevalence in the middle-aged general population increased by 12% (4.8% with 2021 definition), HFmrEF increased by 54% (2.12% with 2021 definition), and HFpEF decreased by 11% (2.19% with 2021 definition) [47]. Previous reviews by Savarese et al. [113], Groenewegen et al. [11], and Dunlay et al. [12] also observed wide variations in HFmrEF/HFpEF prevalence across countries, and a decline in HFpEF incidence was observed, although the results were relatively old, with the most recent incidence results being from 2015.

HFmrEF/HFpEF is associated with considerable mortality. However, HFmrEF/HFpEF exhibits varying mortality rates because of factors like study design, follow-up duration, patient characteristics, treatment approaches, and HFmrEF/HFpEF definitions, making it hard to derive a range. The mortality risk can differ for various types of HF. HFmrEF tends to have lower all-cause mortality compared to HFpEF, potentially due to its lower risk characteristics, as explained by Jentzer et al. [73]. In another study, patients with HFmrEF share more similar characteristics with HFrEF than with HFpEF, yet HFpEF and HFmrEF still exhibit comparable mortality rates, both of which are lower than the mortality rates seen in HFrEF [1]. This may be due to their higher LVEF; previous studies have indicated that recovery from a reduced LVEF is linked to better outcomes [114–117]. In a study by Borlaug et al., HFpEF and HFrEF patients showed similarly poor survival rates, but differed in causes of death. HFpEF had fewer cardiovascular and more non-cardiovascular deaths compared to HFrEF. This highlights the significance of effectively managing non-cardiac comorbidities in HFpEF [112]. Mortality rates in RWE studies were higher than RCTs because of differences in patient populations. RCTs typically include younger, healthier individuals with fewer underlying health conditions, who are closely monitored in outpatient settings with shorter follow-up periods.

HFmrEF/HFpEF is associated with considerable morbidity and poor reported patient outcomes. Key co-morbidities are well identified in the HFmrEF/HFpEF setting, with the main common ones being hypertension, atrial fibrillation, stroke, diabetes, obesity, COPD, and CKD. Two studies by Chamberlain et al. highlight an association between HF and a higher prevalence of comorbidities, which vary based on HF type, age, and sex [118, 119]. Notably, comorbidities were more common in men, and patients with HFpEF had an additional condition compared with HFrEF (mean: 4.5 vs 3.7). This underscores the importance of considering HF type when addressing comorbidities and tailoring treatment approaches accordingly [119].

Patients with HFmrEF/HFpEF require frequent hospitalizations. Patients with HFpEF face a higher rate of the first hospitalization for HF than patients with HFmrEF (9.6 vs 8.9 per 100 patient-years), while HF hospital readmission rates are similar between HFpEF and HFmrEF (44.6% vs 40.1% for the first readmission and 23.3% for HFmrEF vs 17.1% for the second readmission, respectively) [13, 120]. The high disease morbidity is impacting the HRQoL of patients with HFmrEF/HFpEF. Patients with HFpEF yield poorer PROs, impacting QoL (overall KCCQ scores), compared to those with HFmrEF or HFrEF. This impact is further heightened by frequently associated non-cardiac comorbidities like T2D, CKD, and obesity. In a study by Joseph (2013), NYHA class correlated with KCCQ scores in both HFpEF and HFmrEF groups, suggesting that HRQoL could be related to factors other than EF, such as symptom severity [96].

As a result, early intervention is crucial to prevent disease burden. Optimizing the prevention and treatment of these conditions could potentially prevent a substantial number of HF cases [118]. RCT results suggest that interventions in HFpEF and HFmrEF, particularly SGLT-2is like dapagliflozin and empagliflozin, reduce the risk of CV death or HF hospitalization [79, 80]. Although the mortality reduction was limited, the significant decrease in HF hospitalization risk shows promise for the improvement of HFmrEF/HFpEF management and outcomes [79, 80]. Treatments for HFpEF and/or HFmrEF demonstrated promising safety profiles overall, with specific side effects observed for some drugs, such as hypotension and hyperkalemia for sacubitril/valsartan [32, 80]. More research in real-world settings is needed to better understand the safety implications, especially in the long term. Some studies have found positive effects on HRQoL with SGLT-2is, particularly dapagliflozin [80] and empagliflozin [78]. However, sacubitril/valsartan did not significantly impact HRQoL [32]. Spironolactone showed short-term improvements in patient-reported outcomes but not in the longer term [98]. Although some KCCQ scores reached statistical significance, the clinical relevance of the differences was uncertain, as indicated in regulatory reports. The mean differences in KCCQ scores for both SGLT-2is and sacubitril/valsartan were not clinically meaningful. For empagliflozin, the change in the KCCQ clinical summary score from the baseline at week 52 was statistically significant but modest, raising doubts regarding its clinical relevance [102, 121, 122]. The proportion of patients achieving a clinically relevant change (5 points) was slightly higher in the empagliflozin group (41.7%) compared with the placebo group (38.7%), but the difference in the percentage was small. Other KCCQ scores also showed treatment differences, although they were, again, considered small and not clinically relevant [123].

Data on costs and resource utilization in patients with HFmrEF/HFpEF are scarce. Nonetheless, this TLR highlights the increasing economic burden. High healthcare costs per patient and rising hospitalizations call for more cost-effective management strategies. On the other hand, the available data are primarily limited to the United States, so future research should explore the global economic impact and assess the long-term effects of different management approaches.

Although treatments for HFrEF are established and effective, until recently, there was an important evidence gap in relation to therapeutic options that provide significant benefits for patients with HFmrEF/HFpEF. Three new drugs have entered the HFpEF space since 2020, with promising data from their clinical trials. Sacubitril/valsartan was approved in patients with HF and a below normal LVEF based on the PARAGON-HF trial [10]. Later, empagliflozin was approved for HFmrEF/HFpEF in 2022 based on data from the EMPEROR-Preserved trial [6, 7, 9], with dapagliflozin arriving on the market in 2022/2023 as a result of data obtained from the DELIVER trial [8, 9]. Real-world studies indicate that the utilization of SGLT-2is has thus far been limited, which may be because of their recent introduction, but this is expected to increase with accumulating evidence. Guidelines are starting to integrate recommendations for SGLT-2is in HFmrEF/HFpEF, driven by emerging evidence. This includes specific recommendations for empagliflozin which are supported by trial outcomes within the HFmrEF/HFpEF population. Notably, organizations such as the American Heart Association (AHA), American College of Cardiology (ACC), and Heart Failure Society of America (HFSA) in 2022 have contributed to these evolving guidelines [2]. According to recent updates, SGLT-2is (empagliflozin and dapagliflozin) were recently recommended by Japanese guidelines for patients with HF regardless of LVEF [124]. More recently, a focused update of the 2021 ESC guidelines was published in August 2023, which also recommends empagliflozin and dapagliflozin for patients with HFmrEF/HFpEF [125].

In addition, there is a growing focus on HFmrEF/HFpEF indication, with four products (tirzepatide [126], semaglutide [127], ziltivekimab [128], and mitiperstat [129]) in phase 3 of development. However, most of these trials are being conducted in restricted populations with specific comorbidities, such as obesity and/or type 2 diabetes [126–129]. Moreover, in May 2023, the FDA approved the first dual SGLT-1 and SGLT-2 inhibitor, sotagliflozin, for the broad treatment of HF, based on phase 3 results from the SCORED trial and the SOLOIST-WHF trial [105, 130].

Despite the introduction of new drugs, unmet medical needs remain and new therapeutic options for HFmrEF/HFpEF are required, because there has been no demonstrably clear effect on mortality in dedicated HFmrEF/HFpEF trials (CV death or all-cause mortality), the change in KCCQ total symptom score is not deemed clinically meaningful, and there are specific safety warnings for gliflozins.

This review’s inclusion of a wide range of publications, including RCTs, RWE publications, and guidelines from various locations, ensures a comprehensive and up-to-date summary of the published literature in this field. However, this TLR has some limitations, including the choice of the TLR over the SLR methodology, potentially affecting the comprehensiveness of the findings, and the study prioritization process, which might have excluded some relevant studies. Nevertheless, this is balanced by the assurance that crucial data were obtained by examining recent reviews/SLRs from the past 2–3 years concerning the same subject. Finally, publication bias and geographical restrictions may have influenced the generalizability of the findings.

## Conclusions

HFmrEF and HFpEF present a meaningful and growing burden on the global healthcare system. Recent advances have improved our understanding of the epidemiology, pathophysiology, and diagnosis of these conditions, along with the approval of drugs offering promising treatment options. However, there remain key knowledge gaps in terms of the burden of illness and unmet medical needs requiring alternative treatment approaches. Further research and efforts are needed to address these gaps and develop more effective strategies to manage and improve outcomes for patients with HFmrEF/HFpEF.

### Supplementary Information

Below is the link to the electronic supplementary material.

Supplementary file1 (DOCX 51 KB)

## Data Availability

This manuscript has data included as supplementary material.

## Footnotes

1 Authors analyzed the KCCQ total symptom score as a composite outcome based on the rank of the change in score from baseline to moth 8, with a corresponding win ratio used to estimate the magnitude of the treatment effect.
