# Supplementary material for: Heart failure with mildly reduced and preserved ejection fraction: A review of disease burden and remaining unmet medical needs within a new treatment landscape
Source: Heart Fail Rev. 2024 Feb 27;29(3):631–62. doi: 10.1007/s10741-024-10385-y (PMC11035416; doi:10.1007/s10741-024-10385-y)
Supplement: Supplementary file 1 — Supplementary file1 (DOCX 51 KB) [file 10741_2024_10385_MOESM1_ESM.docx]

## Supplementary tables

Supplementary Table 1. Search strategy for online databases using hand searches

| **Scope** | **Source/database** | **Link (access via)** |
| --- | --- | --- |
| **Treatment guidelines** |  | |
| **France** | French National Authority for Health (HAS, Haute Autorité de Santé) | [www.has-sante.fr](http://www.has-sante.fr) |
| **Sweden** | The National Board of Health and Welfare (Socialstyrelsen) | <https://www.socialstyrelsen.se/> |
|  | Swedish Medicines Agency (Läkemedelsverket) | <https://lakemedelsboken.se/kapitel/hjarta-karl/hjartsvikt.html> |
|  | Knowledge management health care (Kunskapsstyrning hälso- och sjukvård) | <https://kunskapsstyrningvard.se/kunskapsstyrningvard.44259.htm> |
|  | Tandvårds- och läkemedelsförmånsverket (TLV) | <https://www.tlv.se/> |
|  | Regional: Region Stockholm | <https://janusinfo.se/>  <https://kunskapsstodforvardgivare.se/> |
| **Germany** | National Care Guideline (NVL) | <https://www.leitlinien.de/> |
|  | The German Society of Cardiology (DGK) | <https://leitlinien.dgk.org/> |
|  | Deutsches Ärzteblatt International | <https://www.aerzteblatt.de/> |
|  | Institut für Qualität und Wirtschaftlichkeit im Gesundheitswesen (IQWiG) | [www.iqwig.de](http://www.iqwig.de) |
| **United Kingdom** | Guidelines (Summarizing clinical guidelines for primary care) | <https://www.guidelines.co.uk/> |
|  | National Institute for Health and Care Excellence (NICE) | <https://www.nice.org.uk/> |
|  | British Cardiovascular Society | <https://www.bsh.org.uk/> |
| **United States** | American Heart Association (AHA) | <https://www.heart.org/> |
|  | Heart Failure Society of America (HFSA) | <https://hfsa.org/> |
|  | American College of Cardiology (ACC) | <https://www.acc.org/> |
| **Japan** | The Japanese Circulation Society (JCS) | <https://www.j-circ.or.jp/english/> |
|  | The Japanese Heart Failure Society (JHFS) | <http://www.asas.or.jp/jhfs/english/> |
|  | Japanese College of Cardiology | <http://www.jcc.gr.jp/en/index.html> |
| **Europe** | The European Society of Cardiology (ESC) | <https://www.escardio.org/> |
|  | Heart Failure Association of the ESC | <https://www.escardio.org/Sub-specialty-communities/Heart-Failure-Association-of-the-ESC-(HFA)/News> |
| **Other (incl. international/global)** | Guidelines International Network (GIN) | <https://g-i-n.net/> |
|  | Agency for Healthcare Research and Quality (AHRQ) | <https://www.ahrq.gov/gam/index.html> |
|  | Heart Rhythm Society (HRS) | <https://www.hrsonline.org/> |
| **Regulatory websites (labels/current competitive landscape)** | | |
| **Europe** | European Union (European Medicines Agency, EMA) | <https://www.ema.europa.eu/en> |
| **United Kingdom** | Medicines and Healthcare products Regulatory Agency, MHRA | <https://products.mhra.gov.uk/> |
| **United States** | Food and Drug Administration, FDA | <https://www.fda.gov/> |
| **Japan** | Pharmaceuticals and Medical Devices Agency, PMDA | <https://www.pmda.go.jp/english/> |
| **Clinical trials registries** | |  |
| **Global** | ClinicalTrials.gov database | <https://clinicaltrials.gov/> |

Abbreviations; HAS, French National Authority for Health; TLV, Dental and Pharmaceutical Benefits Agency; NVL, National Care Guideline; GDK, German Society of Cardiology; IQWiG, Independent Institute for Quality and Efficiency in Health Care; NICE, National Institute for Health and Care Excellence; AHA, American Heart Association; HFSA, Heart Failure Society of America; ACC, American College of Cardiology; JCS, Japanese Circulation Society; JHFS, Japanese Heart Failure Society; ESC, European Society of Cardiology; GIN, Guidelines International Network; AHRQ, Agency for Healthcare Research and Quality; HRS, Heart Rhythm Society; EMA, European Medicines Agency; FDA, Food and Drug Administration; MHRA, Medicines and Healthcare products Regulatory Agency; PMDA, Pharmaceuticals and Medical Devices Agency.

Supplementary Table 2. Eligibility criteria for targeted literature review

| **PICOS** | **BOI —Eligibility criteria and restrictions** | |
| --- | --- | --- |
| **Population** | Adult patients with HF (NYHA II-IV) and LVEF ≥40% | |
| **Intervention/Comparator** | No restrictions | |
| **Outcomes** | **Epidemiology, clinical and humanistic burden:**  •Definition, diagnosis (diagnostic code)  •Incidence  •Prevalence  •Mortality  •Co-morbidity (including DM, AF, renal insufficiency/renal failure, sleep apnea)  •CV death  •HF events (HHF or urgent HF visit)  -Total HF events  -Recurrent HF events  •Improvement in NYHA class  •QoL  •Renal events^a^  •Hospitalizations (all-cause, CV)  •Non-fatal CV events (including non-fatal MI, non-fatal stroke, HHF)  •Change in UACR from baseline  •Days alive and out of hospital  •New onset of atrial fibrillation | **Treatment overview:**  •Treatment options  •Treatment pattern/practice  •Percentage of patients receiving each treatment type  •Adherence/compliance  •Discontinuation rate/AEs^b^  •Predictors and risk factors for HF  •Risk scores  •Unmet needs  **Economic burden:**  •Resource use  •Direct costs  •Indirect costs  **Economic evaluations:**  •Model structure and methods  •Input data with key sources  •Results of model |
| **Study design** | RWE^c^, RCT, treatment guidelines, systematic reviews, CEA, BIA  Not restricted for economic burden | |
| **Year** | From 2012 to present (epidemiology, economic burden, RWE) | |
| **Countries** | US, Europe, Japan (epidemiology, guidelines, treatment patterns) | |
| **Additional restrictions** | English language (not restricted for treatment guidelines) | |

Abbreviations: AEs, adverse events; AF, atrial fibrillation; BIA, budget impact analysis; CEA, cost-effectiveness analysis; CV, cardiovascular; DM, diabetes mellitus; HF, heart failure; HHF, hospitalization due to heart failure; HTA, health technology assessment; LVEF, left ventricular ejection fraction; NYHA, New York Heart Association; QoL, quality of life; RCT, randomized controlled trial; RWE, real-world evidence; UACR, urine albumin-creatinine ratio; US, United States.

^a^sustained decrease in estimated glomerular filtration rate (eGFR) ≥50%, sustained decrease in eGFR ≥57%, sustained eGFR decline to <15 ml/min/1.73m2, initiation of dialysis or renal transplantation.

^b^Safety outcomes: Kidney injury/acute kidney injury, ketoacidosis, bladder cancer, amputation, hyperkalemia, gynecomastia, genital infections, urosepsis, hospitalization for hyperkalemia, severe hyperkalemia, hypotension, urinary infections, pyelonephritis, hyponatremia, hypoglycemia, fractures, volume depletion, diabetic ketoacidosis, worsening of renal function, a number of subjects discontinuing permanently or temporarily due to hyperkalemia or worsening of renal function, anemia, stroke, and syncope.

^c^Excluding letters, comments, case series, case studies, reviews.

Supplementary Table 3. Overview of clinical guidelines with a focus on heart failure with preserved ejection fraction/heart failure with mildly reduced ejection fraction

| **Location** | **Short guideline title & date** | **Use of an evidence-grade approach** | **Definition  (based on LVEF)** | | **Diagnosis criteria (general)** | | **Specific diagnostic algorithm/risk score** | | **Elements of dedicated disease management** | | **Potential limitations of current medications as per guidelines** | | **NT proBNP value threshold** | |
| --- | --- | --- | --- | --- | --- | --- | --- | --- | --- | --- | --- | --- | --- | --- |
|  |  |  | **HFmrEF** | **HFpEF** | **HFmrEF** | **HFpEF** | **HFmrEF** | **HFpEF** | **HFmrEF** | **HFpEF** | **HFmrEF** | **HFpEF** | **HFmrEF** | **HFpEF** |
| **Europe** | ESC 2021[1] | ● | **●** | **●** | **●** | **●** | **○** | **●** | **●** | **●** | **●** | **●** | **●** | **●** |
|  | HFA/ESC 2020[26] | ● | **○** | **●** | **○** | **●** | **○** | **●** | **○** | **●** | **○** | **○** | **○** | **●** |
|  | ESH 2021[30] | ● | **○** | **●** | **○** | **●** | **○** | **○** | **○** | **●** | **○** | **●** | **○** | **●** |
| **France** | HAS 2015[29] | ○ | **○** | **●** | **○** | **○** | **○** | **○** | **○** | **○** | **○** | **○** | **○** | **○** |
|  | HAS 2014[18] | ● | **○** | **●** | **○** | **●** | **○** | **○** | **○** | **●** | **○** | **●** | **○** | **●** |
|  | SFGG 2021[28] | **○** | **●** | **●** | **●** | **●** | **○** | **○** | **○** | **●** | **○** | **●** | **●** | **●** |
| **Germany** | NVL 2019[23] | ● | **●** | **●** | **●** | **●** | **○** | **○** | **●** | **●** | **●** | **●** | **●** | **●** |
|  | DGK 2021 [17] | ● | **●** | **●** | **●** | **●** | **○** | **●** | **●** | **●** | **○** | **●** | **●** | **●** |
|  | NDMG 2018 [27] | ● | **●** | **●** | **○** | **○** | **○** | **○** | **●** | **●** | **○** | **○** | **○** | **○** |
|  | IQWiG 2021 [19] | ● | **●** | **●** | **○** | **○** | **○** | **○** | **○** | **○** | **○** | **○** | **○** | **○** |
| **Sweden** | NBHW 2018 [21] | ● | **○** | **○** | **○** | **○** | **○** | **○** | **○** | **○** | **○** | **○** | **○** | **○** |
|  | LOK 2022 [20] | **○** | **●** | **●** | **○** | **○** | **○** | **○** | **●** | **○** | **○** | **●** | **○** | **○** |
|  | SMA 2015 [25] | ● | **○** | **●** | **○** | **●** | **○** | **○** | **○** | **●** | **○** | **●** | **○** | **●** |
|  | SKS 2021 [24] | ● | **●** | **●** | **○** | **○** | **○** | **○** | **●** | **●** | **●** | **●** | **○** | **○** |
| **United Kingdom** | NICE 2018 [22] | ● | **○** | **○** | **○** | **○** | **○** | **○** | **○** | **○** | **○** | **○** | **○** | **○** |
|  | CaReMeUK-HF 2022 [31] | ○ | **○** | **○** | **○** | **○** | **○** | **○** | **○** | **●** | **○** | **○** | **○** | **○** |
| **United States** | AHA/ACC/HFSA 2022 [2] | ● | **●** | **●** | **●** | **●** | **○** | **●** | **●** | **●** | **●** | **●** | **●** | **●** |
| **Japan** | JCS/JHFS 2021 [3] | ● | **●** | **●** | **○** | **●** | **○** | **●** | **●** | **●** | **○** | **●** | **○** | **○** |

Abbreviations: HFmrEF, heart failure with mildly reduced ejection fraction; HFpEF, heart failure with preserved ejection fraction; HAS, French National Authority for Health; TLV, Dental and Pharmaceutical Benefits Agency; NVL, National Care Guideline; DGK, German Society of Cardiology; IQWiG, Independent Institute for Quality and Efficiency in Health Care; NICE , National Institute for Health and Care Excellence; AHA, American Heart Association; ACC , American College of Cardiology; JCS , Japanese Circulation Society; JHFS, Japanese Heart Failure Society; ESC, European Society of Cardiology; G-BA, Federal Joint Committee; ESH, European Society of Hypertension; SFGG, French Society of Geriatrics and Gerontology; NDMG, National Disease Management Guideline; NBHW, The National Board of Health and Welfare; LOK, Pharmaceutical committees' national network; SMA, Swedish Medicines Agency; SKS, national system for knowledge management Health care Sweden's regions in collaboration; CaReMeUK, British Cardiovascular Society; HF, Heart Failure; LVEF, left ventricular ejection fraction;

● The guideline contains recommendations on the respective aspect of care.
○ The guideline does not contain any recommendations on the respective aspect of care.
